# Human brain networks function in connectome-specific harmonic waves

**DOI:** 10.1038/ncomms10340

**Published:** 2016-01-21

**Authors:** Selen Atasoy, Isaac Donnelly, Joel Pearson

**Affiliations:** 1 School of Psychology, University of New South Wales, Sydney, New South Wales 2052, Australia; 2 School of Mathematics and Statistics, University of New South Wales, Sydney, New South Wales 2052, Australia

## Abstract

A key characteristic of human brain activity is coherent, spatially distributed oscillations forming behaviour-dependent brain networks. However, a fundamental principle underlying these networks remains unknown. Here we report that functional networks of the human brain are predicted by harmonic patterns, ubiquitous throughout nature, steered by the anatomy of the human cerebral cortex, the human connectome. We introduce a new technique extending the Fourier basis to the human connectome. In this new frequency-specific representation of cortical activity, that we call ‘connectome harmonics', oscillatory networks of the human brain at rest match harmonic wave patterns of certain frequencies. We demonstrate a neural mechanism behind the self-organization of connectome harmonics with a continuous neural field model of excitatory–inhibitory interactions on the connectome. Remarkably, the critical relation between the neural field patterns and the delicate excitation–inhibition balance fits the neurophysiological changes observed during the loss and recovery of consciousness.

## Abstract (web summary)

Inter-areal oscillatory synchronization is constrained by anatomical connections, yet a fundamental principle linking functional and structural connectivity is lacking. Here, Atasoy and colleagues show that harmonic patterns in structural connectome can predict dynamics of resting state networks.

## Graphical abstract

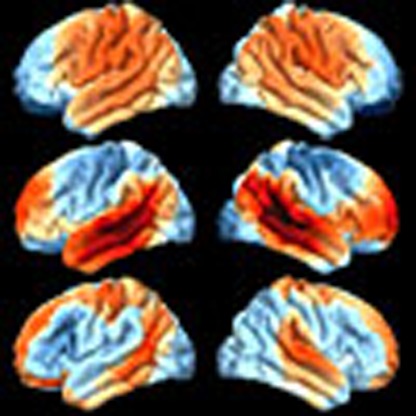

Fundamentals of natural processes are expressed by physical laws, quantitative relationships among measured properties that are always true^1^. Although the discovery of the mechanisms of neuronal excitation provided a milestone in explaining single neuron behaviour^2^, a fundamental principle underlying the collective neural dynamics has remained largely elusive^3^.

A characteristic feature of cortical dynamics in mammals is the emergence of behaviour-dependent oscillatory networks spanning five orders of magnitude in the frequency domain^4^. Recently, strong temporal correlation within widely distributed cortical regions has also been discovered in spontaneous slow (<0.1 Hz) fluctuations of the blood oxygen level-dependent signal measured with functional magnetic resonance imaging (fMRI). This discovery revealed that spontaneous activity, in the absence of any external stimuli or task condition, also exhibits highly structured correlation patterns throughout the brain. Remarkably, the topography of these correlation patterns, termed the resting state networks (RSNs)^5^,^6^, closely resembles the functional networks of the human brain identified by various sensory, motor and cognitive paradigms^6^,^7^ and have been found to relate to electroencephalography microstates, global brain states occurring in discrete epochs of about 100 ms (refs 8, 9).

The RSNs are thought to emerge from local cortical dynamics and cortico-cortical interactions constrained by the anatomical structure of the human cortex—the human connectome^10^,^11^. Indeed, various computational models have explored the spontaneous emergence of such oscillatory networks from anatomical connectivity, local cortical dynamics and cortico-cortical interactions^10^,^12^. However, our understanding of neural activity lacks a unified fundamental principle revealing a direct macroscopic description of the collective cortical dynamics^3^.

Here, we demonstrate that a ubiquitous mathematical framework, eigendecomposition of the Laplace operator, which lies at the heart of theories of heat, light, sound, electricity, magnetism, gravitation and fluid mechanics^13^, can predict the collective dynamics of human cortical activity at the macroscopic scale. In various natural phenomena, the eigenfunctions of the Laplacian constitute the basis of self-organizing patterns in a system: standing wave patterns emerging in sound-induced vibrations of a guitar string or a metallic plate (first demonstrated as complex sand patterns by Chladni^14^), patterns of ion motion emerging from electromagnetic interactions^15^,^16^, electron wave function of a free particle given by time-independent Schrödinger equation^17^,^18^ and even patterns emerging in complex dynamical systems such as the reaction-diffusion models introduced by Turing^19^, which can explain various instances of biological pattern formation^20^, are predicted by the eigenfunctions of the Laplace operator^21^,^22^ (Fig. 1a). Furthermore, Laplace eigenfunctions computed on one-dimensional domain with periodic boundary conditions such as a circle, correspond to the well-known Fourier basis^23^. This relation has been utilized to extend the Fourier transform to more complex geometries^23^ and to define a ‘shape DNA'^24^ in shape recognition.

We investigate the extension of Laplace eigenfunctions to the particular structure of the human connectome, the connectome harmonics, as a new representation for macroscale cortical activity. Remarkably, when described in this new connectome-specific extension of the Fourier basis, the RSNs match the spatial patterns (Laplacian eigenfunctions) corresponding to certain natural frequencies (Laplacian eigenvalues). Our results present evidence that Laplace eigenfunctions can provide a simple yet almost universal description for patterns of synchrony throughout the cortex in the resting state. Further, we demonstrate a plausible biological mechanism behind the emergence of these patterns from the cortico-cortical and thalamo-cortical interactions by modelling the excitatory and inhibitory dynamics with a neural field model.

## Results

### Connectome harmonics predict resting state networks

**Figure 1:**
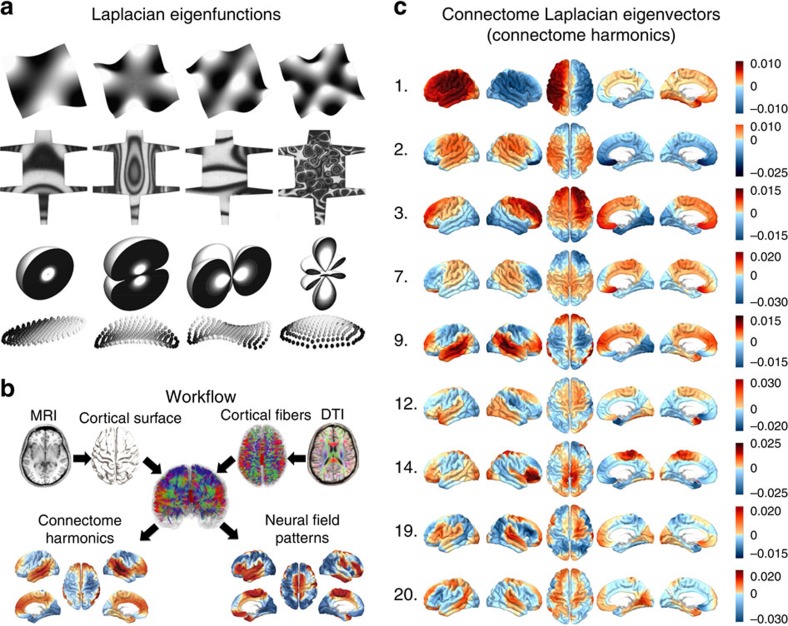
Laplace eigenfunctions and connectome harmonics. (**a**) Laplace eigenfunctions revealing the mechanical vibrations of rectangular metal plates (1st row)—first demonstrated by Ernst Chladni as patterns formed by sand on vibrating metal plates—and metal plates shaped as mammalian skin (2nd row) resembling different mammalian coat patterns for different frequency vibrations^21^,^22^ (images reprinted from^22^ with permission) as well as electron orbits of the hydrogen atom computed by time-independent Schrödinger's wave function (3rd row)—shown with increasing energy from left to right—and patterns emerging in electromagnetic interactions between laser-excited ion crystals (last row) (images adapted from^15^). (**b**) Workflow for the construction of macroscale connectome model. The graph representation was formed by connecting each node sampled from the cortical surface with its immediate local neighbours and by further including the long-range connections between the end points of the cortico-cortical and thalamo-cortical fibres. (**c**) Examples from the 20 lowest frequency connectome harmonics. Left: wave number. Right: spatial patterns of synchronous oscillations estimated by the eigenvectors of the connectome Laplacian.

To define the extension of the Laplace operator applied to the human connectome, we utilized its discrete counterpart, the graph Laplacian. We first created a graph representation for each of 10 human connectomes by combining the cortical surface extracted from MRI data of 10 subjects with the long-range (white matter) cortico-cortical and thalamo-cortical connections generated from cortical fibre tracts derived from diffusion tensor imaging (DTI) data of the same subjects (Fig. 1b). For each subject, we formed the graph representation 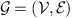 of the modelled connectome, where the nodes 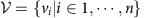
−*n* being the total number of nodes—uniformly sample the curved anatomy of the cortical surface and the edges 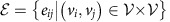 incorporate both local and long-range cortico-cortical and thalamo-cortical connections. By computing the graph Laplacian 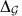 on the introduced representation 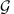, we define the discrete counterpart of the Laplace operator applied to the human connectome, the connectome Laplacian, for each individual and estimate its eigenvalue–eigenvector pairs 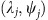, 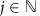, connectome harmonics (see Methods). It is worth noting that by harmonics here we refer to spatial harmonics—as opposed to temporal harmonics. We will see later that these spatial harmonics can emerge from neuronally plausible dynamics but, at this stage, we are basing our Laplace eigenfunctions on static structural connectomes. It is also important to note that the introduced graph 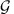 differs from previous graph representations of the human connectome used in population models^10^ in that it does not incorporate any parcellation of the cortical surface and cortico-cortical and thalamo-cortical connections. Thus, it provides a densely sampled connectome model with the minimum amount of discretization possible in the given resolution of the MRI and DTI data. Notably, when the number of uniformly sampled data points taken from the underlying manifold, such as the cortical surface, increases, the graph Laplacian converges to its continuous counterpart, the Laplace–Beltrami operator—the generalization of the Laplacian to non-euclidean geometries such as the curved anatomy of the human cortex^25^.

In the literature, Laplace eigenvalue–eigenvector pairs (eigenmodes) have received significant attention initially due to their relation to the excitation spectrum of a given geometry: the eigenvalues relate to the natural frequencies, the allowed frequencies of standing waves emerging on that particular geometry, whereas the eigenvectors yield the associated wave patterns^18^,^23^. Recent studies demonstrate also the relevance of Laplace eigenmodes for other physical phenomena including the phase extraction of an electron wave function^18^, the patterns emerging in electromagnetic interactions of ions^15^,^16^ and morphogenesis^22^,^26^ (Fig. 1a). Here, we utilized the eigenvectors of the connectome Laplacian to describe the spatio-temporal patterns of macroscale neural activity.

We found that the eigenvectors of the connectome Laplacian, the connectome harmonics, yield frequency-specific spatial patterns across distributed cortical regions (Fig. 1c; Supplementary Fig. 1). Here the red–blue patterns represent examples from the first 20 connectome harmonics in ascending order of frequency (wavenumber—shown in left) for one representative subject.

**Figure 2:**
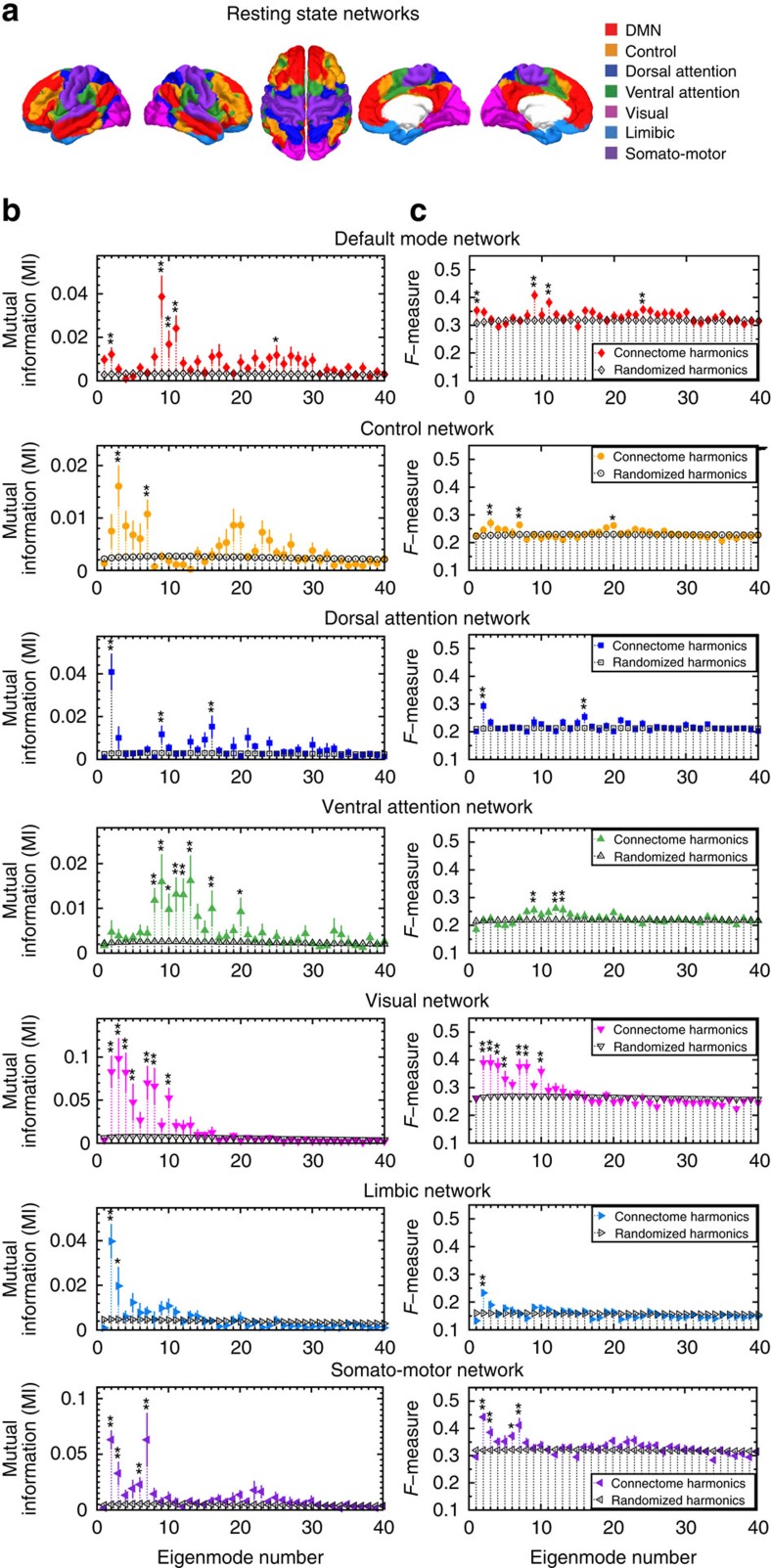
Prediction of the RSNs by connectome harmonics. (**a**) Patterns of synchronous oscillations, i.e., the RSNs, of the human brain overlap with the established functional systems, i.e., groups of cortical regions, which coactivate during certain tasks^6^,^7^. For quantitative evaluation of any similarity between the RSNs and connectome harmonics, we use the seven RSNs (default mode, control, dorsal attention, ventral attention, visual, limbic and somato-motor networks) (shown in **a**) identified from 1,000 subjects' intrinsic functional connectivity data^27^. (**b**) Similarity measured by mutual information and (**c**) predictive power measured by *F*-measure values between the connectome harmonics with 40 lowest frequencies and the reference RSNs in ref. 27 (shown in **a**) compared with those of randomized harmonics (*P<0.0002, **P<0.0001 estimated by Monte Carlo simulations with 2,000 simulations per subject and 500,000 simulations for group average, after multiple comparison correction by false discovery rate, error bars indicate standard error across 10 subjects).

To quantitatively evaluate any similarity between the connectome harmonics and seven RSNs (Fig. 2a), commonly observed in the human brain; default mode, control, dorsal attention, ventral attention, visual, limbic and somato-motor network, we measured the mutual information between the reference RSNs^27^ and each of the connectome harmonic patterns (Fig. 2b: filled, coloured data points). As a control, we performed Monte Carlo simulations (2,000 simulations per subject randomly combined to 500,000 simulations for group average), in which we randomized the long-range white-matter cortico-cortical and thalamo-cortical connections while preserving the local anatomical structure of the subject's cortical surface and computed the harmonics of each randomized network using the same methodology (Fig. 2b: black data points). Crucially, we found statistically significant similarity between the default mode network (DMN) and one particular connectome harmonic (in the range of the 9th connectome harmonic, ±2 due to individual differences) in the group average (Fig. 2b, top row, *P<0.0002, **P<0.0001; 500,000 Monte Carlo simulations for group average, corrected for multiple comparisons) as well as for all 10 subjects individually (Supplementary Fig. 2, *P*-values: *P<0.05, **P<0.02; 2,000 Monte Carlo simulations per subject, corrected for multiple comparisons). For all other resting state networks, we observed larger individual differences in the mutual information values (Supplementary Figs 2 and 3), while the Monte Carlo simulations still yielded significant similarity for different ranges of the connectome harmonics in the group analysis (Fig. 2b). In particular, we found that the visual, somato-motor and limbic networks showed significant similarity to only low-frequency connectome harmonics, while networks associated with higher cognitive functions, that is, control, dorsal attention and ventral attention, significantly matched a broad range of connectome harmonics distributed over the spatial frequency spectrum.

To further assess the predictive power of connectome harmonics for the resting state networks, we utilized an information retrieval metric, *F*-measure, which simultaneously quantifies the recall and precision of connectome harmonics' prediction of the RSNs. We computed *F*-measure values between the connectome harmonics (after applying a binary indicator function) and the binary patterns of the RSNs^27^ and compared it with the *F*-measure values of randomized harmonics derived from Monte Carlo simulations. The *F*-measure provides a stricter evaluation than the information theoretic mutual information, as the indicator function imposes a binary decision to each node. This evaluation (Fig. 2c; Supplementary Figs 4 and 5) confirmed our previous findings based on mutual information. Taken together, these results suggest that the visual, somato-motor, and limbic, as well as the default mode network, are well predicted by individual connectome harmonics of a narrow frequency range, whereas the higher cognitive networks rely on a broader frequency range of connectome harmonics distributed over the spatial spectrum.

### Connectome harmonics as the Fourier basis on the connectome

Next, we investigated the use of connectome harmonics as a function basis to represent spatial patterns of cortical networks. The orthogonality of connectome harmonics means that a linear combination of these eigenfunctions can be used to recreate any spatial pattern of neural activity. The importance of the connectome harmonic basis lies in its close ties to the classical Fourier transform, which corresponds to the decomposition of a signal into a linear combination of the eigenfunctions of the Laplace operator applied to a circular domain, that is, sine and cosine functions with different frequencies^23^. Since connectome harmonics are defined as the eigenvectors of the connectome Laplacian—the discrete counterpart of the Laplacian applied to the human connectome—they extend the Fourier basis to the particular geometry of the human connectome. Hence, the spectral transform onto the connectome harmonic basis naturally extends the classical Fourier transform to the human connectome.

**Figure 3:**
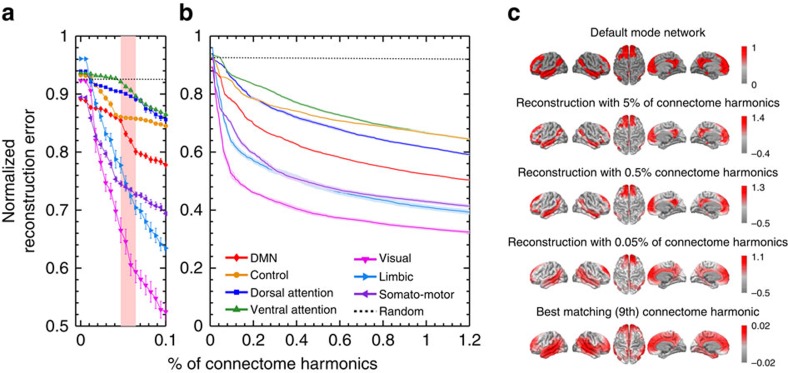
Reconstruction of the RSNs from connectome harmonic basis. Normalized reconstruction error of each resting state network using (**a**) 0.1% and (**b**) 1.2% of the connectome harmonics spectrum averaged across 10 subjects (error bars and shading indicate standard error across 10 subjects) compared to the reconstruction of a randomized binary pattern. Red band in **a** highlights the steepest decrease for the DMN. (**c**) Reconstruction of the DMN using (from top to bottom) 5, 0.5 and 0.05% of the spectrum and the best matching connectome harmonic of one subject's data.

To analyse the spatial frequency content of the resting state networks^27^ (Fig. 2a), we performed a spectral transform to the connectome harmonic basis and reconstructed the spatial patterns of individual networks. Although the binary nature of the reference networks theoretically necessitates the use of the whole connectome harmonic spectrum for reconstruction—the same way that a square wave can only be reconstructed using the sine waves with infinite many frequencies—sharp decreases in the normalized reconstruction errors were observed by using just 0.1% of the connectome harmonic spectrum (low-frequency range; Fig. 3a). The steepest decrease of the DMN's reconstruction error occurred for the frequency band that also showed significant similarity and predictive power in mutual information and *F*-measure values, respectively (highlighted by the red column in Fig. 3a, best matching connectome harmonic of each subject shown in Supplementary Fig. 6, reconstruction shown in Fig. 3c) while for the visual, somato-motor and limbic networks the decrease of the reconstruction error remained large but constant within 0.1% of the spectrum (Fig. 3a). Slower convergence was observed for the reconstruction errors of higher cognitive networks within the range of 1.2% of the spectrum (low-frequency range) suggesting the reliance of these networks on a broader range of frequencies (Fig. 3b). These results confirm our previous findings while providing a novel analytical language of cortical activity analogous to the classical Fourier transform that can be utilized to quantify any activity pattern including task-based event-related designs.

### Biological mechanisms underlying connectome harmonics

We also investigated the biological mechanisms likely to underlie the self-organization of connectome harmonics on the cortex. Hitherto, we have assumed that the graph Laplacian based upon structural connectivity provides a plausible proxy for the effective connectivity (also known as the Jacobian-please see ref. 28) of an underlying neuronal dynamical system. In what follows, we explore the biological mechanisms likely to underlie these neuronal dynamics. In particular, we exploit the efficient description of these dynamics given by neural field equations in terms of connectome harmonics and show how they give rise to the emergence of connectome harmonics on the cortex.

The dynamics of the oscillatory cortical networks is thought to emerge from the interplay of excitation; for instance mediated by glutamatergic principal cells, and inhibition; for instance mediated *γ*-aminobutyric acid GABAergic interneurons^29^. To describe macroscale cortical dynamics, we extend a variant of neural field models based on Wilson–Cowan equations, the most commonly utilized mathematical description of the excitatory–inhibitory neural dynamics^30^. Neural field models based on Wilson–Cowan equations are a variant of reaction-diffusion systems^31^ originally introduced by Turing as a mathematical model for morphogenesis^19^. Based on the principle that mutual interactions between a diffusing activator and inhibitor can result in self-organizing pattern formation, reaction-diffusion models have provided valuable insights into the mechanisms underlying the emergence of non-linear waves in several biological processes including morphogenesis^19^,^20^, formation of ocular dominance patterns in the visual cortex^32^, visual hallucinations^33^,^34^ and interactions of excitatory and inhibitory activity in neural populations of the cortical and thalamic tissue^30^,^35^. Crucially, the pattern formation in reaction-diffusion systems is caused by the exponential growth of certain eigenfunctions of the Laplacian applied to the patterning domain (see refs 21, 22 and Supplementary Notes 1–4). The selection of which harmonic patterns are ‘activated' (grow) is determined by the diffusion parameters of excitation and inhibition (Supplementary Fig. 7). Hence, the Laplace eigenfunctions provide the building blocks of complex patterns emerging in reaction-diffusion systems.

On a two-dimensional continuous idealization of the cortex, the Wilson–Cowan equations lead to self-organization of neural oscillatory patterns when short-range excitation is coupled with broader lateral inhibition^36^. This type of functional circuitry, known as ‘Mexican hat' organization^37^ or centre-on and surround-off connectivity^38^ is well-observed experimentally in the early visual cortex, known as cortical surround suppression^39^,^40^ and is likely to extend throughout the neocortex^41^,^42^.

Recent experimental evidence showed that a plausible mechanism underlying the cortical surround suppression in V1 is the activity of the somatostatin-expressing inhibitory neurons in the superficial layers of the mouse visual cortex and a similar neural circuit is also likely to underlie surround suppression in other cortical areas^42^. These findings are further supported by the report of broader spatial extent of inhibition compared with excitation in the primary visual cortex of awake mice^43^. Furthermore, layer-specific suppression and facilitation also generates the necessary circuits for lateral inhibitory interactions^29^,^44^: coordinated modulation of superficial (L2/3) and deep cortical layers (L5) gives rise to competition between neighbouring domains and lateral inhibition, although the spatial extent of excitation and inhibition across cortical domains show overlapping distributions vertically (across cortical layers) and horizontally (within layers)^44^. Notably, it has been shown that the Mexican hat type of functional circuitry can also be generated in anatomical circuits with short-range inhibition when a fraction of the total excitatory conductance is slower than the inhibition^37^. A potential biological source of slower excitatory conduction is the slow synaptic transmission caused by the *N*-methyl-D-aspartate receptors contributing mostly to excitatory currents^37^.

Taken together, this converging evidence suggests that various biological mechanisms can give rise to the functional circuitry equivalent to short-range excitation coupled with broad inhibition, which indeed is the necessary condition for the self-organization of oscillatory patterns in Wilson–Cowan-type neural field models^30^,^33^,^35^ (Supplementary Notes 1–5). Next, we extend the Wilson–Cowan equations to the full structural connectivity of the thalamo-cortical system by incorporating the connectome Laplacian and demonstrate the relation between the emerging oscillatory patterns and the connectome harmonics.

### Neural field model of connectome-wide neural dynamics

**Figure 4:**
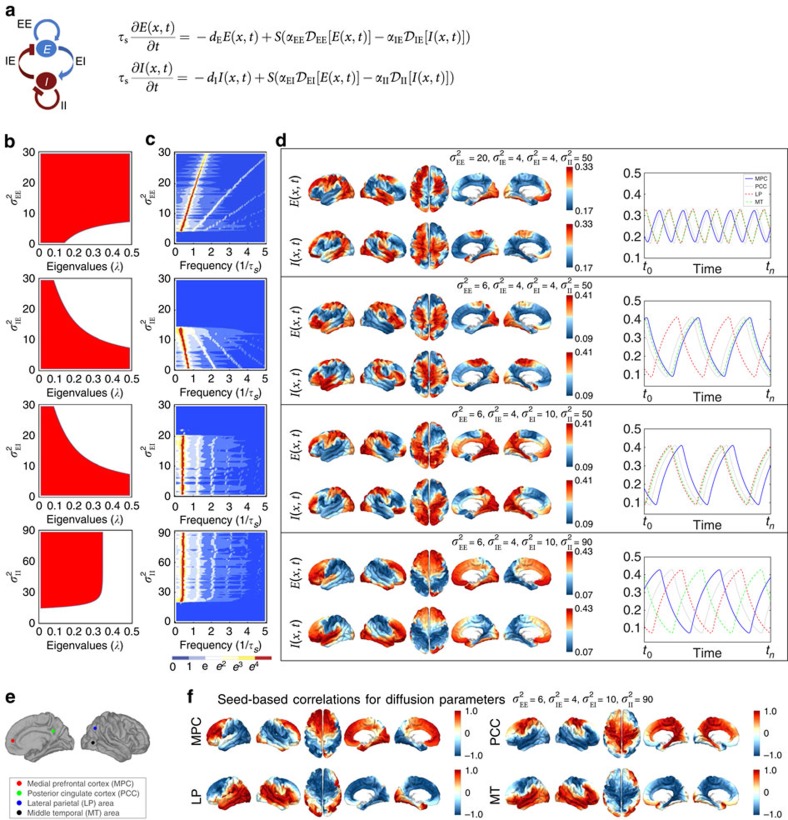
Neural field model. (**a**) Left: dynamics of excitatory (*E*) and inhibitory (*I*) activity. Right: time evolution of the excitatory E(*v*_*i*_, *t*) and inhibitory I(*v*_*i*_, *t*) activity at the cortical location 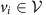 at time *t* where 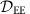, 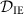, 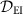 and 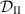 describe the diffusion processes of E and I activity acting on excitatory (EE, IE) and inhibitory (EI, II) neural populations and *τ*_s_ refers to the units of system time, that is, characteristic time scale. (**b**) Linear stability analysis of the neural field model in terms of connectome harmonics. The red regions correspond to the diffusion parameters in the phase space that algebraically satisfy the necessary condition for oscillations, that is, the critical Hopf regime, plotted as a function of the analysed diffusion parameter vertical axis and the eigenvalue of the connectome harmonic horizontal axis. (**c**) Power spectrum of the temporal oscillations in (a total of 267) numerical simulations averaged over all nodes. (**d**) Spatial pattern for an arbitrary time slice and the temporal profile of four seed locations shown in **e**. (**f**) Seed-based correlation analysis of the neural field patterns demonstrates the decoupling between the posterior and anterior midline nodes of the DMN for the same set of parameters leading to slow cortical oscillations.

We extended a variant of the neural field model^35^ based on the Wilson–Cowan equations^30^ to the three-dimensional connectome model by incorporating the connectome Laplacian into the spatial propagation (diffusion) term (Fig. 4a; Supplementary Notes 2 and 3). Numerical simulations were then performed by combining the network diffusion on the human connectome—modelled by iterative application of symmetric graph Laplacian^23^,^45^ 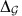
—with the excitatory–inhibitory reaction dynamics (Fig. 4a). This allows us to extend continuous form neural field models by incorporating the connectivity of the human connectome. This neural field approach differs from previous macroscale simulations^10^,^46^ in that spatial propagation is modelled by network diffusion, as opposed to discrete coupling, yielding a (spatially) near-continuous model of cortical dynamics.

We found that structured oscillatory patterns naturally self-organize on the human connectome for a wide-range of diffusion parameters in the model (Fig. 4a–d; Supplementary Figs 8 and 9; Supplementary Movies 1–4). Linear stability analysis of the neural field model revealed that a wide range of connectome harmonics could be activated for different diffusion speeds of excitation and inhibition (Fig. 4b; Supplementary Notes 3–5), rendering the neural field model a plausible neural mechanism for the self-organization of connectome harmonics. In particular, we observed a decrease in the frequency of coherent oscillations when excitatory activity is decreased, modelled by slower diffusion of excitation, or when inhibitory activity is increased, modelled by faster diffusion of inhibition in the neural field model (Fig. 4c,d). This relationship between the frequency of temporal oscillations and the excitation–inhibition balance shows remarkable overlap with the neurophysiological changes observed during the loss and recovery of consciousness (for the analysed parameter range). Neurophysiological evidence suggests that drug- or sleep-induced loss of consciousness is associated with increasing inhibitory or decreasing excitatory activity, which is accompanied by a transition from the low amplitude, high-frequency patterns to low-frequency coherent oscillations in cortical activity^47^. Recent work also shows gradual decoupling between the posterior and anterior midline nodes of the DMN during loss of consciousness^48^,^49^. We observed this decoupling in seed-based correlation analysis of the neural field patterns for the exact parameters, which resulted in slower cortical oscillations (Fig. 4e,f; Supplementary Fig. 9).

**Figure 5:**
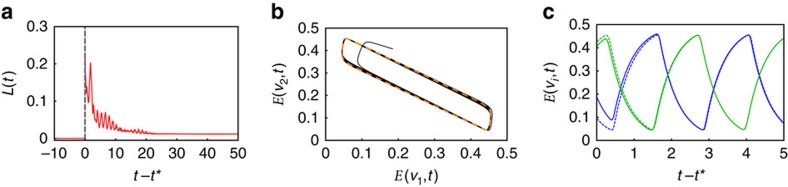
Stability analysis of the neural field model. Stability of the emerging oscillations to external perturbation is tested by perturbing the neural field model for a sample oscillatory parameter set (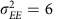, 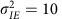, 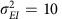 and 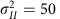). (**a**) The distance measure *L*(*t*) versus the time from perturbation *t*−t* where t* denotes the time of perturbation by white noise. Before the perturbation *L*(*t*) is identical to 0. *L*(0) shows how far the perturbed system altered from the underlying oscillatory stable state. After perturbation, that is, when *t*−t*>0, *L*(*t*) approaches 0 showing the system is Lyapunov stable. (**b**) The convergence of the limit cycle; that is, excitatory activity of two non-adjacent nodes plotted over time, of the perturbed (black solid line) to unperturbed (orange dashed line) system. (**c**) The original (unperturbed) trajectory (dashed line) and the perturbed trajectory (solid line) for two sample vertices (green and blue lines) after perturbation *t*−t*∈[0, 5] unit time. The perturbed trajectory converges back to the original trajectory (with a small phase shift) demonstrating that the state corresponding to the original trajectory is Lyapunov stable to small perturbations.

Finally, we tested the stability of the emerging oscillatory patterns to external perturbations such as noise using Lyapunov stability analysis^50^. This method involves perturbing the system at some time t* and observing whether the perturbed system converges to the original system. In dynamical systems, such as described by neural field models, the eigenvalues of the linearized system around a known fixed point immediately reveals the local behaviour of the system; that is, it would allow one to identify the unstable ‘growing' eigenmodes that dominate observed fluctuations (Supplementary Notes 3–5). However, due to the nonlinearities inherent in the Wilson–Cowan equations as well as the high dimensionality of the modelled system, the continuum of the fixed points, that is, the trajectory, was not readily obtainable analytically. Therefore, we numerically integrated the system to determine the stable solution, in our case a periodic solution, for certain parameters. We first perturbed the system separately 10 times by white noise at time t* and analysed the largest difference between the unperturbed trajectory and each of the perturbed trajectories over time by computing the *L*-infinity norm *L*(*t*) (see Methods). Fig. 5a shows that *L*(*t*) is bounded and converges to *ɛ*≈0, whereas Fig. 5b,c illustrate the convergence of the limit cycle and the temporal oscillations of the perturbed system to that of the unperturbed system for two example vertices *v*_1_ and *v*_2_. These results demonstrate that for the analysed parameter range the extended Wilson–Cowan equations (Fig. 4a) are robust to external perturbations such as noise.

Taken together, the extension of the Wilson–Cowan-type neural field models to the particular structural connectivity of the human connectome provides a biologically plausible, robust mechanism likely to underlie the self-organization of connectome harmonics in the thalamo-cortical system.

## Discussion

Our results reveal several notable findings: firstly, by extending a universal mathematical framework, eigendecomposition of the Laplacian, to the anatomical structure of the human connectome, we introduce connectome harmonics—a connectome-specific extension of the Fourier basis—as a new representation to describe and analyse any spatio-temporal patterns of cortical activity. Remarkably, decomposition of the RSNs into the connectome harmonics revealed significant overlap between the resting state networks and certain connectome harmonic patterns. This suggests that connectome harmonics provide a simple explanatory principle linking the dynamics of oscillatory cortical networks to the anatomy of the human connectome. This finding is the first experimental evidence demonstrating that the ubiquitous mathematical framework, eigendecomposition of the Laplacian, when applied to the human connectome, can provide a simple yet almost universal principle possibly underlying the functional networks of the brain.

Secondly, due to the orthogonality, and therefore independence, of the connectome harmonics corresponding to different frequencies (wavenumbers), the set of all connectome harmonics provides a function basis (a new coordinate system or representation) to describe and analyse any spatio-temporal pattern of cortical activity, independent of the imaging modality, experimental design and even species. Although in this work we demonstrate the application of this novel technique for the human connectome, it can be extended to any other mammalian brain, given its structural connectivity. Hence, connectome harmonics provide a new frequency-specific language to describe spatio-temporal patterns of neural activity and open the door to a new dimension of tools available to probe brain dynamics across various species and technologies.

The potential importance of having an efficient basis set (connectome harmonics) may be particularly relevant for dynamic causal modelling. Indeed, the eigenfunctions of the graph Laplacian are used to summarize the activity of cortical patches in dynamic causal models of electromagnetic activity^51^. From the point of view of dynamic causal modelling for resting state fMRI, the eigenmodes of the functional connectivity have been used as prior constraints on effective connectivity. Our work suggests that these could be replaced with the eigenfunctions of the graph Laplacian of the structural connectome. Furthermore, one could adjudicate between the utility of eigenmodes based on structural and functional connectivity using Bayesian model comparison—that would compromise a further validation of our universal basis set.

Thirdly, we have emphasized the universality of eigenfunctions or eigenmodes of the graph Laplacian, connectome harmonics, in describing coupled dynamical systems; such as neural field models. By using the graph Laplacian defined on the human connectome, we demonstrate how well-studied neural field models such as those based on the Wilson–Cowan equations^30^ can be extended to the full structural connectivity of the thalamo-cortical system and linked to the RSNs of the human brain. However, as the definition of connectome harmonics is independent of the chosen dynamical model, both analytically and numerically, the introduced mathematical framework of connectome harmonic basis can also be applied to extend other more complex network models; (for example refs 10, 12, 52), to the full structural connectivity of the thalamo-cortical system in (spatially) near-continuous domain.

In fact, recent computational models suggest very rich and complex dynamics, characterized by rapid transitions between a few discrete states of correlated activity can emerge in resting state^52^, which is initiated by the noise-driven exploration of the repertoire of the correlation states^10^,^12^. These computational studies are supported by recent empirical evidence showing complex dynamics between different RSNs. Studies combining the high temporal resolution of magnetoencephalography data with band-width-specific correlation analysis suggest that the RSNs assemble and disassemble over time, enabling communication (through periods of coherent oscillations) not only within but also across different networks^53^,^54^,^55^. Furthermore, fMRI studies using sliding time window correlations instead of averaging correlations over the whole time course found that a discrete set of connectivity states, dominant recurrent patterns of correlated neural activity, emerge and dissolve over tens of seconds^56^,^57^. Connectome harmonic basis could provide a novel theoretical framework for linking these recent empirical findings and advanced computational models focusing on noise-driven exploration of the connectivity states, as they reveal the patterns of correlation on the thalamo-cortical system, and define the set of independent connectivity states or building blocks through which complex dynamics can be expressed in a non-parametric manner, for instance without tuning the size of the temporal window for correlation analysis^58^,^59^. Hence, re-formulating the dynamical models, such as those in ref. 52, in terms of connectome harmonics could reveal how noise-driven transitions occur between different frequencies and results in communication between different RSNs.

An intuitive understanding of the role of connectome harmonics in describing coupled dynamical systems, in the setting of large-scale brain dynamics, rests on associating the graph Laplacian with the effective connectivity among cortical nodes. In other words, if the graph Laplacian corresponds to a matrix of effective connection strengths, then its eigenfunctions become the eigenmodes or principle components of the functional connectivity. Furthermore, the graph Laplacian or effective connectivity becomes the (negative) partial correlation matrix of observed fluctuations in activity^28^. This is important because it explains why the eigenmodes based on the graph Laplacian provide a plausible space in which to describe the RSNs based on functional connectivity. This correspondence rests upon undirected connectivity (represented by symmetric adjacency) matrices, which means that one can interpret effective connectivity as mediating a ‘diffusion' of neuronal activity. To complete this picture, note that the diagonal terms of the graph Laplacian ensure the sum of input connections to any node is zero (see Methods). It is important to note, however, that in contrast to the diffusion process in other physical phenomena, the neural activity propagates not only locally but also through the long-range white-matter thalamo-cortical connections, the ‘diffusion' process occurs on the particular connectivity of the human connectome. Our description of the universal harmonics implied by the graph Laplacian in terms of diffusion rests on the undirected nature of the structural connectome (represented by a symmetric adjacency matrix). However, we know that reciprocal forward and backward connections show strong asymmetries in the human brain, rendering the conceptual link between the (directed) effective connectivity and diffusion not always valid. Having said this, there is no reason why one cannot pursue modelling and simulation using the eigenmodes of directed effective connectivity matrices^28^.

In summary, in this work we introduce a new connectome-specific representation of cortical activity patterns and dynamics, which extends the Fourier basis to the structural connectivity of the thalamo-cortical system. Remarkably, when expressed in this new analytic language, RSNs of the human brain overlap with the connectome harmonic patterns of certain frequencies. We demonstrate the self-organization of these connectome-specific harmonics patterns from the interplay of neural excitation and inhibition in coupled dynamical systems as described by neural field models. Interestingly, due to the emergence of these harmonic patterns in various natural phenomena, ranging from acoustic vibrations, electromagnetic interactions and electron wave functions to morphogenesis, it is tempting to suppose that human brain activity might also be governed by the same underlying principles as other natural phenomena.

## Methods

### Data

Data used in the preparation of this work were obtained and made available by the Human Connectome Project (HCP), WU-Minn Consortium (Principal Investigators: David Van Essen and Kamil Ugurbil; 1U54MH091657), which is funded by the 16 NIH Institutes and Centers that support the NIH Blueprint for Neuroscience Research and by the McDonnell Center for Systems Neuroscience at Washington University. We use MRI and DTI data of 10 unrelated subjects (six female, age 22–35) provided by the HCP, WU-Minn Consortium, available on https://db.humanconnectome.org/data/projects/HCP_500. All MRI and DTI datasets were preprocessed according to minimal preprocessing guidelines of the HCP protocol and no additional preprocessing was performed.

For quantitative evaluation of the resting state networks (RSNs) we use the parcellation of the cerebral cortex into seven networks (default mode, control, dorsal attention, ventral attention, visual, limbic and somato-motor networks) identified from 1,000 subjects' intrinsic functional connectivity data^27^, available on http://surfer.nmr.mgh.harvard.edu/fswiki/CorticalParcellation_Yeo2011.

### Workflow

From T1-weighted MRI data of each subject (resolution 0.7 mm), we reconstruct the cortical surfaces separating the white and grey matter, referred to as the white matter surface in the rest of the manuscript, as well as grey matter and the cerebrospinal fluid, referred to as the grey matter surface in the rest of the manuscript, separately for each hemisphere using the Freesurfer Software http://freesurfer.net. We register each cortical surface to the 1,000 subject average cortical surface of the cortical parcellation data (in the rest of the manuscript referred to as average subject, represented by 20,484 vertices), in order to allow for the vertex-to-vertex comparison between the connectome harmonics and the 1,000 subject averaged reference of the resting state networks.

From the DTI data of each subject (resolution 1.25 mm), we extract the white matter cortico-cortical and thalamo-cortical fibres by applying a deterministic tractography algorithm^60^ using the MATLAB implementation of Vista Lab, Stanford University http://white.stanford.edu/newlm/index.php/MrDiffusion. After registering the DTI data and the cortical surface of each subject, we initialize the seeds for tractography on the cortical surface separating the white and grey matter. Centred around each vertex (node)—in total 20,484—we initialize eight seeds and perform the tractography with the following parameters: fractional anisotropy threshold 0.3, that is, fractional anisotropy <0.3 being termination criteria for the tracking, minimum tract length 20 mm, and maximum angle between two contiguous tracking steps 30°.

### Graph representation of the human connectome

The graph representation of the human connectome 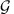 is formed by representing the vertices sampled form the surface of grey matter by the nodes 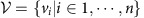 with *n* being the total number of nodes (20,484 in this study) and by including the local and long-range connections between the vertices as the edges 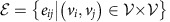 of the graph 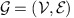. Thereby, the local connections correspond to the connections of the vertices on the cortical surface mesh (six connections per vertex to their nearest neighbours) and the long-range connections are determined by the white-matter cortico-cortical and thalamo-cortical fibres. To this end, the nearest vertex of each fibre end point is identified on the grey matter cortical surface and a long-range connection between the two vertices is added for each fibre tract. In this study, we use an undirected, unweighted graph representation leading to the following adjacency matrix:


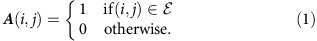


It is important to note that this graph model of the human connectome differs from the previous studies^10^,^12^, in which each node represents one cortical region acquired by a certain parcellation of the cerebral cortex and the edges denote the fibre density between the cortical regions. In contrast to previous graph models of the human connectome, the particular representation used in our study closely approximates the continuous form of the human cortex due to the uniform and dense sampling of the vertices (nodes) from the continuous grey matter cortical surface.

### Connectome Laplacian and connectome harmonics

Given the graph representation 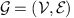 of the human connectome, we compute the symmetric graph Laplacian 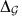 on the connectome graph in order to estimate the discrete counterpart of the Laplace operator^23^,^45^ Δ applied to the human connectome, the connectome Laplacian, as:


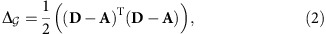


where the adjacency matrix **A** is defined in equation (1) and


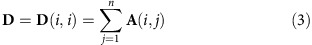


denotes the degree matrix of the graph. We then calculate the connectome harmonics 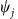, *j*∈{1, ⋯, *n*} by solving the the following eigenvalue problem:


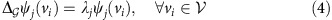


with *λ*_*j*_, *j*∈{1, ⋯, *n*} being the corresponding eigenvalues of 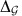.

### Neural field model

Let *E*(*x*, *t*) and *I*(*x*, *t*) correspond to the activity; that is, local spatio-temporal averages of membrane-potentials, of the excitatory and inhibitory neurons at the cortical location 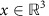 at time *t*. Following the Wilson–Cowan equations^30^,^35^, time evolution of the excitatory and inhibitory neural firing rates satisfy the following non-linear differential equations^35^:


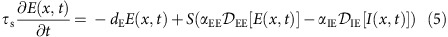



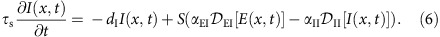


where 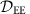, 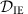, 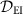 and 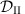 denote the diffusion (spatial propagation) operators of excitatory (*E*) and inhibitory activities (*I*), each separately acting on excitatory and inhibitory populations with names (EE, IE) and (EI, II) respectively. Here *S* denotes the sigmoidal activation function,


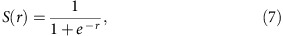


and *τ*_s_ is a characteristic time scale of the system. We extend the Wilson–Cowan equations to the full structural connectivity of the thalamo-cortical system by incorporating the connectome Laplacian to the diffusion (spatial propagation) terms (Supplementary Notes 1 and 2).

### Lypunov stability

We test the robustness of the neural field model for perturbations using Lyapunov stability analysis^50^. This method involves perturbing the system at some time t* and observing whether the perturbed system converges to the original system. If this is the case, the system may be called Lyapunov stable. As the neural field model is oscillatory for the parameter sets we are concerned with, we must define a measure, which determines the distance between two states. We first perturb the system separately 10 times by white noise at time t*. We then take the absolute value of the difference at each node at each time step and taking the maximum difference. This is known as a *L*-infinity norm and is commonly used in stability analysis as it looks at the worst case instead of an average case. At each time step we compare all differences across the 10 perturbed systems and again take the worst case. This quantity is defined to be *L*(*t*). We plot the distance measure *L*(*t*) over time and observe whether *L*(*t*)→0 or *L*(*t*)→*ɛ* with *ɛ*≈0. In Fig. 5(a) we see that *L*(*t*)→*ɛ* and is bounded demonstrating the robustness of the neural field model to noise perturbations.

## Additional information

**How to cite this article:** Atasoy, S. *et al.* Human brain networks function in connectome-specific harmonic waves. *Nat. Commun.* 7:10340 doi: 10.1038/ncomms10340 (2016).

## Supplementary Material

Supplementary Information Supplementary Figures 1-10, Supplementary Table 1, Supplementary Notes 1-5, Supplementary Methods, and Supplementary References

Supplementary Movie 1 Numerical simulations of the neural field equation for excitatory activity on the graph representation of the human connectome for the diffusion parameters σ EE = 20 , σ EI = 4 ,σ IE = 4 ,σ EE = 50 , where the rest of the parameters were set to the default values( d E = dI = 2 ,α EE =α IE =α EI =α II =150

Supplementary Movie 2 Numerical simulations of the neural field equation for excitatory activity on the graph representation of the human connectome for the diffusion parameters σ EE = 6 , σ EI = 4 ,σ IE = 4 ,σ II = 50 , where the rest of the parameters were set to the default values( d E = dI = 2 ,α EE =α IE =α EI =α II =150)

Supplementary Movie 3 Numerical simulations of the neural field equation for excitatory activity on the graph representation of the human connectome for the diffusion parameters σ EE = 6 , σ EI = 4 ,σ IE =10 ,σ II = 50 , where the rest of the parameters were set to the default values( d E = dI = 2 ,α EE =α IE =α EI =α II =150)

Supplementary Movie 4 Numerical simulations of the neural field equation for excitatory activity on the graph representation of the human connectome for the diffusion parameters σ EE = 6 , σ EI = 4 ,σ IE =10 ,σ II = 90 , where the rest of the parameters were set to the default values( d E = dI = 2 ,α EE =α IE =α EI =α II =150)
