# Supplementary material for: Human brain networks function in connectome-specific harmonic waves
Source: Nat Commun. 2016 Jan 21;7:10340. doi: 10.1038/ncomms10340 (PMC4735826; doi:10.1038/ncomms10340)
Supplement: Supplementary Information — Supplementary Figures 1-10, Supplementary Table 1, Supplementary Notes 1-5, Supplementary Methods, and Supplementary References [file ncomms10340-s1.pdf]

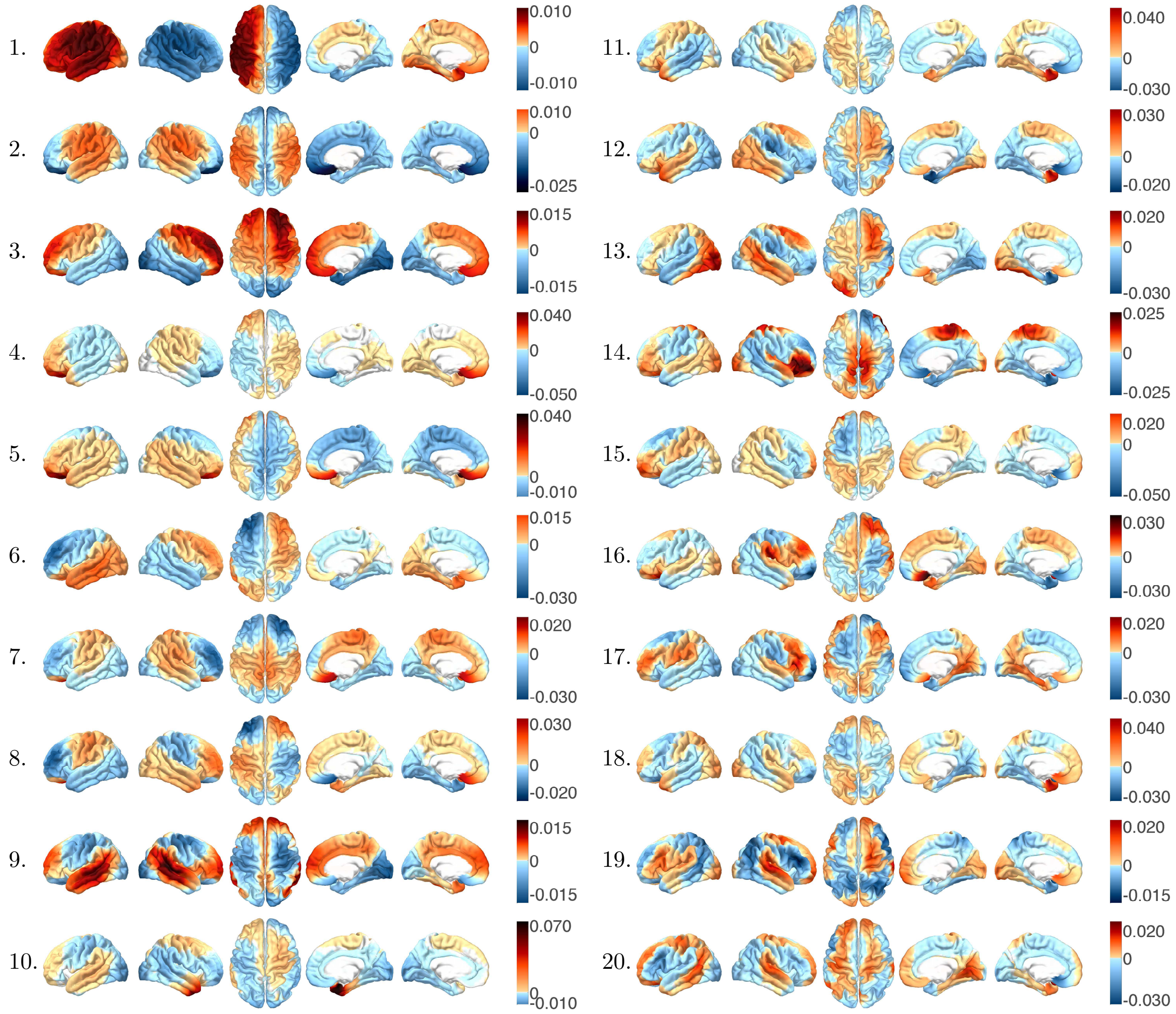

Supplementary Fig. 1: Connectome harmonics of an example subject given by the eigenvectors of the graph Laplacian of the human connectome. Connectome harmonics yield frequency-specific spatial patterns of synchrony across distributed cortical regions shown in ascending order of natural frequency. The color indicates the amplitude of the standing wave and is equivalent to the phase of the oscillation at a particular cortical location for the corresponding natural frequency.

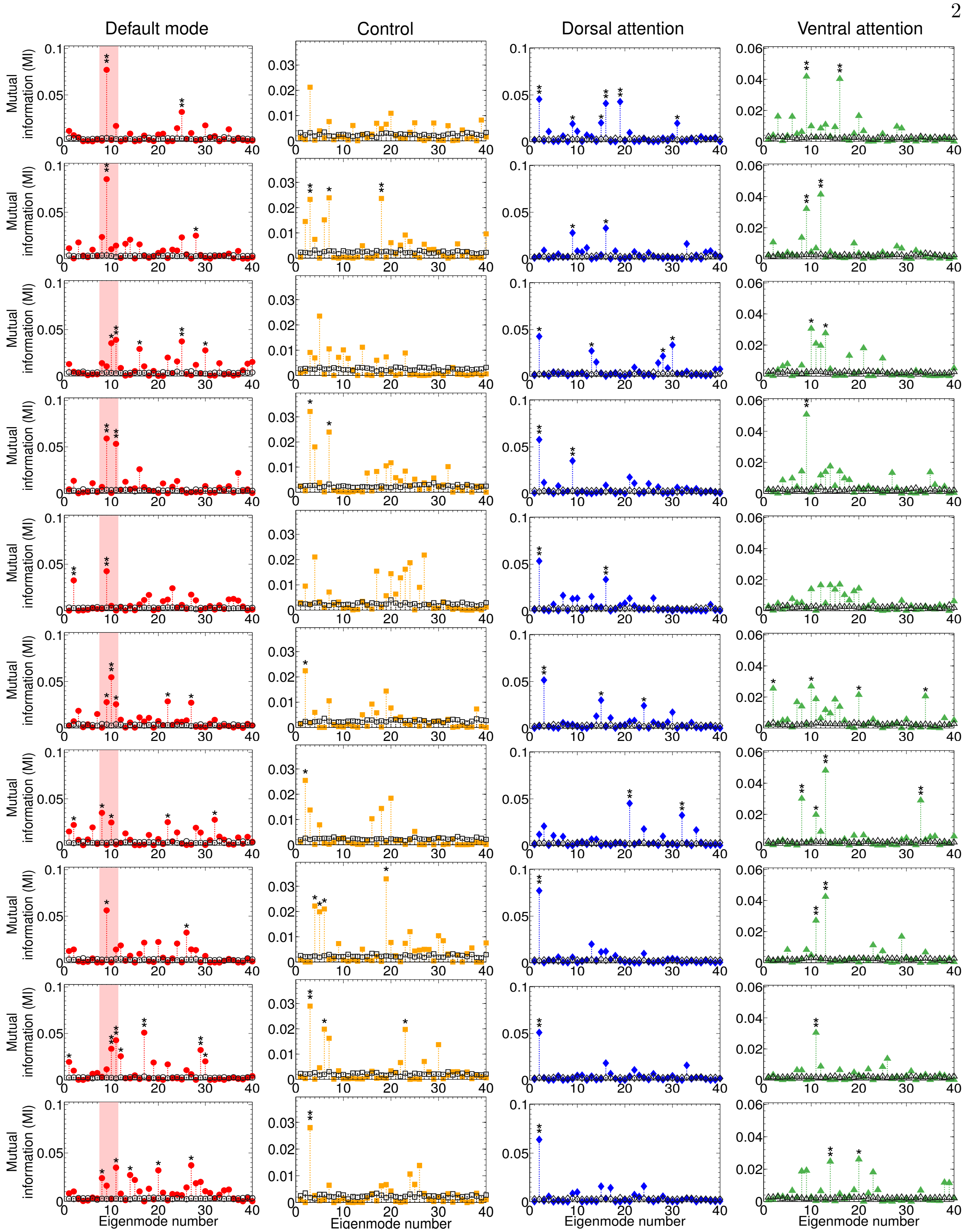

Supplementary Fig. 2: Similarity between the connectome harmonics corresponding to 40 lowest frequencies and the default mode, control, dorsal attention and ventral attention networks in [1]. The similarity is measured by mutual information (displayed as filled color data points) and compared to those of the randomized harmonics (displayed as unfilled black data points). For all 10 subjects statistically significant difference is found between the mutual information values of the connectome and randomized harmonics for the default mode network and one particular connectome harmonic (in the range of the 9th eigenmode, ∓ 2 due to individual differences, highlighted by the red band in the left column, p-values: $* < 0.05$, $** < 0.02$ estimated using Monte Carlo simulations with 2000 randomized trials per subject, after multiple comparison correction for the false discovery rate (FDR)[2]). For control, dorsal attention and ventral attention networks larger individual differences in the mutual information values are observed.

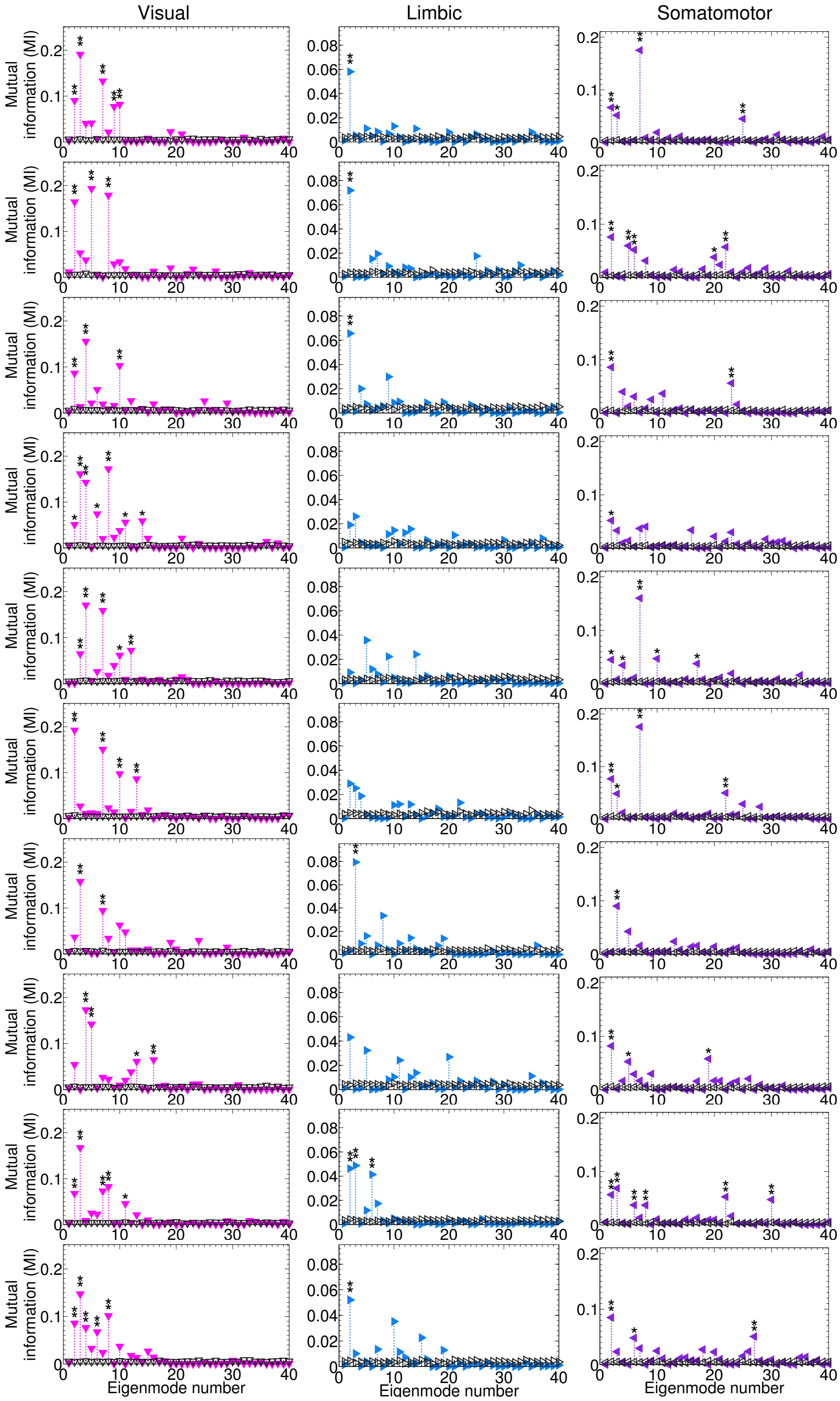

Supplementary Fig. 3: Similarity between the connectome harmonics corresponding to 40 lowest frequencies and the visual, somato-motor and limbic networks in[1]. The similarity is measured by mutual information (displayed as filled color data points) and compared to those of the randomized harmonics (displayed as unfilled black data points). P-values: $* < 0.05$, $** < 0.02$ estimated using Monte Carlo simulations with 2000 randomized trials per subject, after multiple comparison correction for the false discovery rate (FDR)[2]).

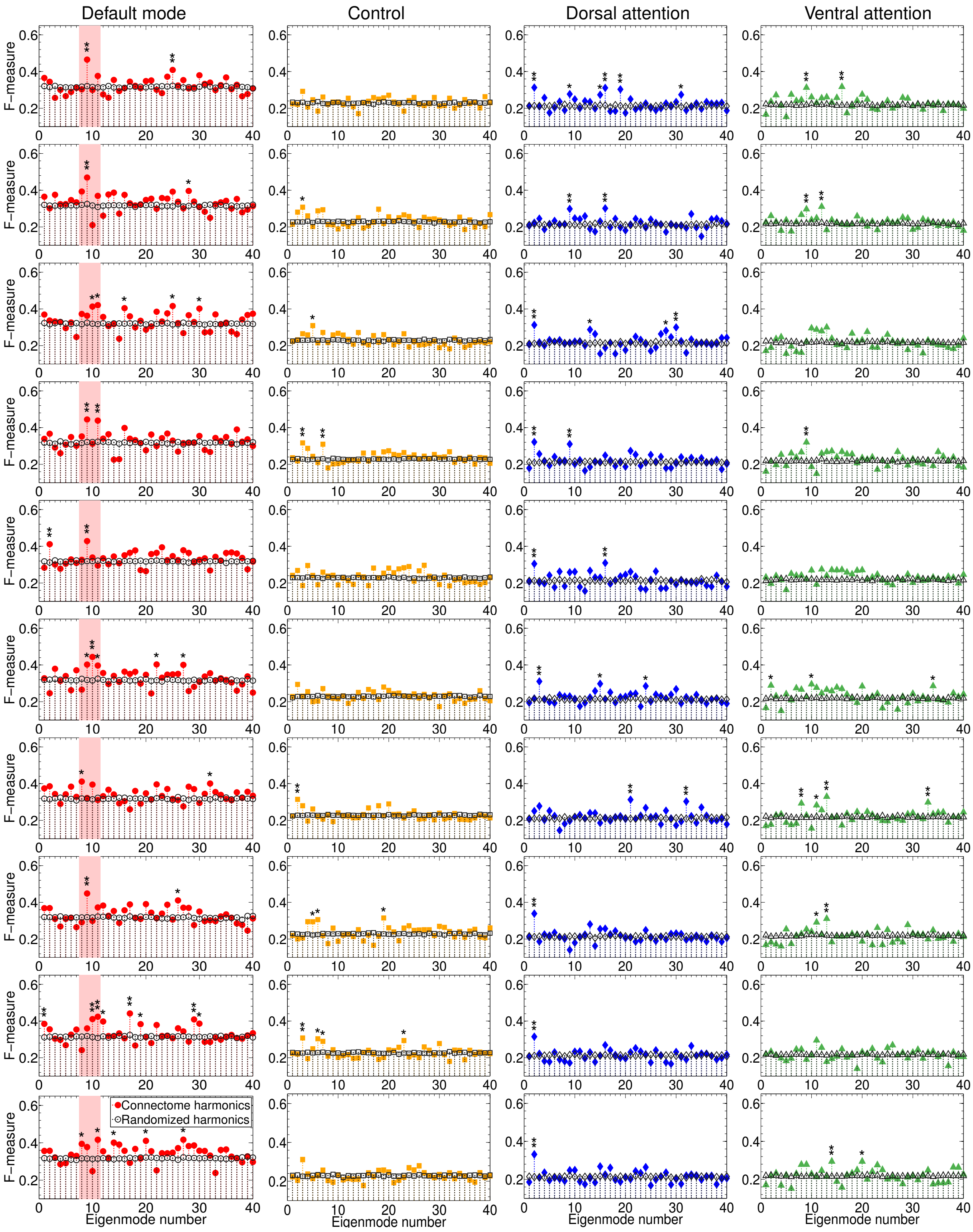

Supplementary Fig. 4: Predictive power of the connectome harmonics corresponding to 40 lowest frequencies and the default mode, control, dorsal attention and ventral attention networks in[1]. The predictive power of connectome harmonics for RSNs is measured by F-measure - harmonic mean of recall and prediction - (displayed as filled color data points) and compared to those of the randomized harmonics (displayed as unfilled black data points). P-values: $* < 0.05$, $** < 0.02$ estimated using Monte Carlo simulations with 2000 randomized trials per subject, after multiple comparison correction for the false discovery rate (FDR)[2]).

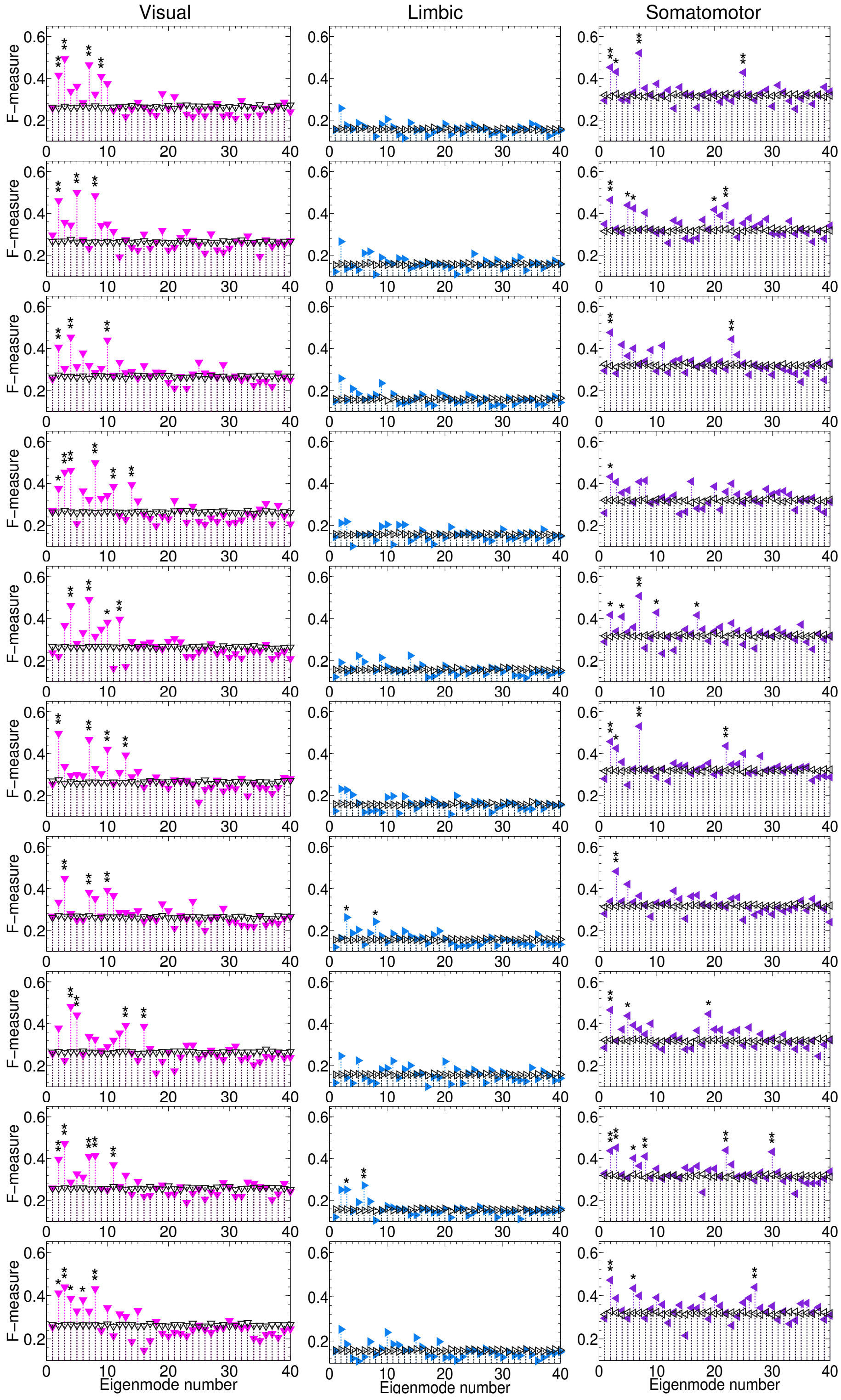

Supplementary Fig. 5: Predictive power of the connectome harmonics corresponding to 40 lowest frequencies and the visual, limbic and somato-motor networks in[1]. The predictive power of connectome harmonics for RSNs is measured by F-measure - harmonic mean of recall and prediction - (displayed as filled color data points) and compared to those of the randomized harmonics (displayed as unfilled black data points). P-values: $* < 0.05$, $** < 0.02$ estimated using Monte Carlo simulations with 2000 randomized trials per subject, after multiple comparison correction for the false discovery rate (FDR)[2]).

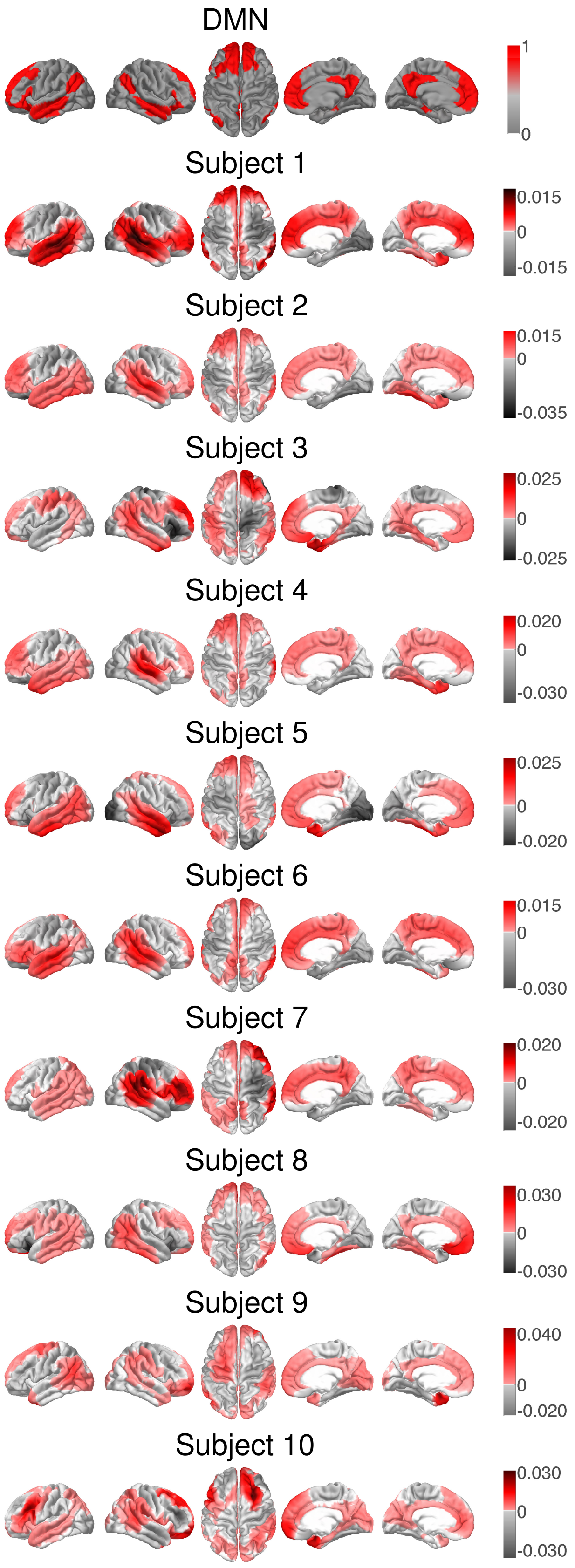

Supplementary Fig. 6: Connectome harmonic patterns of 10 subjects that best match the default mode network (top row).

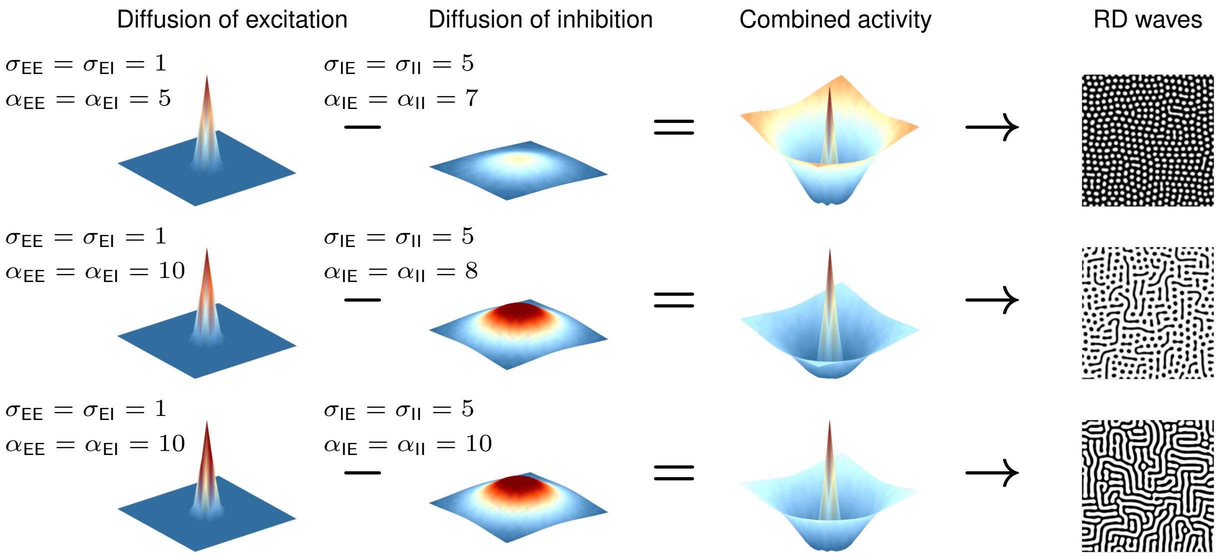

Supplementary Fig. 7: Reaction-diffusion models creating non-linear wave patterns in steady states. Competition between an activator and an inhibitor diffusing at different speeds σ and strength α reacting with each other provides a mechanism for the self-organization of reaction-diffusion waves.

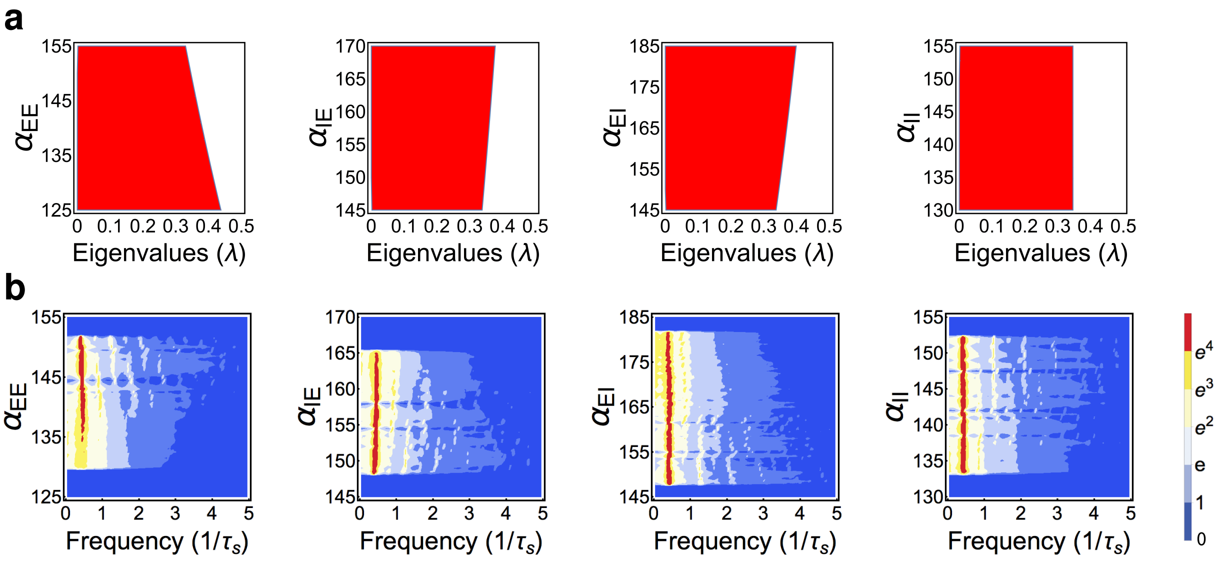

Supplementary Fig. 8: Analysis of the effect of parameter change on frequency of oscillations of the neural field model. (**a**) Linear stability analysis of the neural field model in terms of connectome harmonics. The red regions correspond to the diffusion parameters in the phase space that algebraically satisfy the necessary condition for oscillations, i.e., the critical Hopf regime, plotted as a function of the analyzed diffusion parameter and the eigenvalue of the connectome harmonic. Connectome harmonics within the red region can be activated by the neural field model for the given parameter set (for each plot, α_{EE}, α_{IE}, α_{EI}, α_{II} are varied individually while fixing the remaining parameters to the following default values $d_{\mathsf{E}} = 2$, $d_{\mathsf{I}} = 2$, ${\sigma_{\mathsf{EE}}}^2 = 6$, ${\sigma_{\mathsf{IE}}}^2 = 10$, ${\sigma_{\mathsf{EI}}}^2 = 10$, ${\sigma_{\mathsf{II}}}^2 = 50$. As this is only a necessary, but not sufficient condition, numerical solutions were obtained and the Fourier frequency spectrum from a total of 240 numerical simulations, shown in (**b**). The power of each frequency is the averaged over all nodes in the system. We observe regions where temporal oscillations with certain dominant frequency (red) and its higher harmonics (yellow) are present, i.e., the region satisfies the super-critical Hopf regime, as well as where no oscillations are present (dark blue), i.e., the regions which may satisfy the sub-critical Hopf regime but do not lead to stable oscillatory behavior. However, no significant change in the dominant frequency of temporal oscillations is observed for variations of the $\alpha_{\{\mathsf{EE,IE,EI,II}\}}$ values the analyzed parameter range indicating the change in the temporal oscillations is mainly caused by variations of the diffusion speed, given by the ${\sigma_{\{\mathsf{EE,IE,EI,II}\}}}^2$ values in the neural field model (see Fig. 3 in the main article).

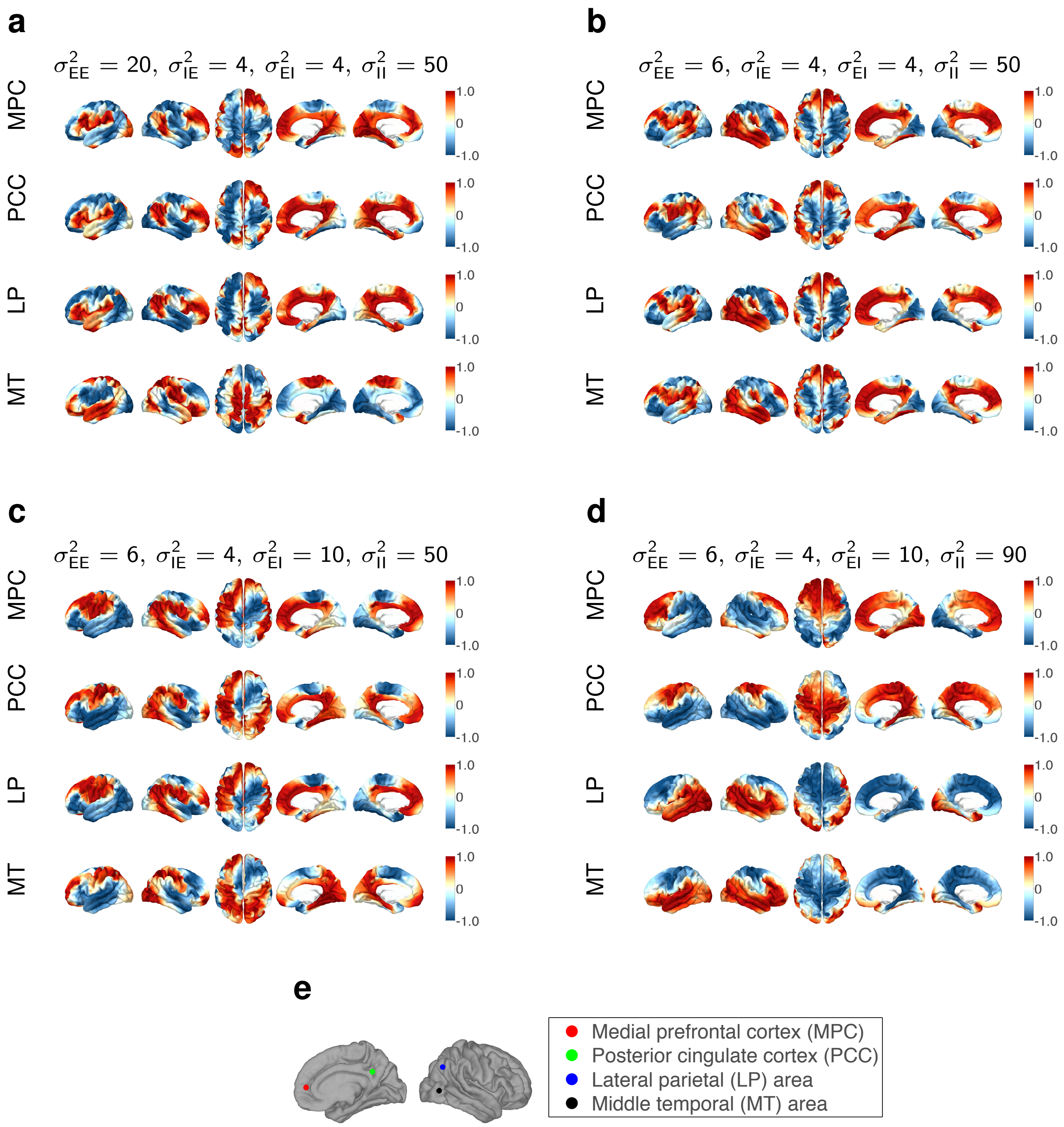

Supplementary Fig. 9: Seed-based correlation analysis of the neural field patterns. (**a**) to (**d**) show the resulting neural field patterns while the excitatory activity is gradually decreased and inhibitory activity is gradually increased. Structured correlation patterns are observed for all 4 set of neural field parameters, where the complexity of the spatial correlation patterns decreases gradually with the decrease of excitation and increase of inhibition from (**a**) to (**d**). For the parameter set yielding low frequency, high amplitude oscillations as observed in loss of consciousness, a decoupling between the anterior and posterior cortex is observed in the correlation patterns (**d**). (**e**) Seed-locations illustrated on the cortex.

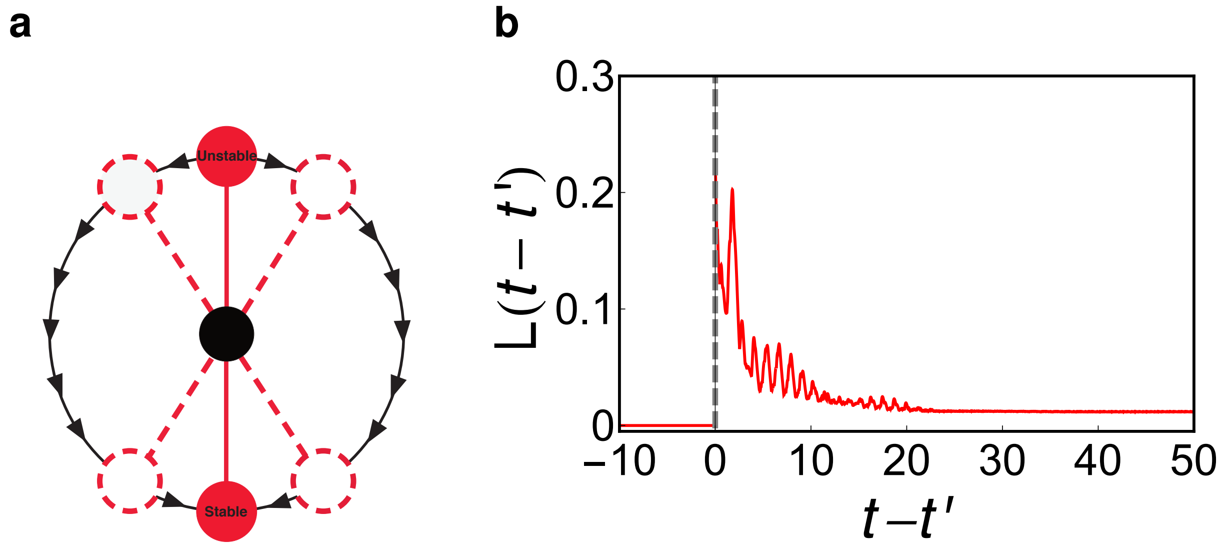

Supplementary Fig. 10: Illustration of stable and unstable states and Lypunov analysis. **(a)** illustrates the stable and unstable states of a system on the pendulum example. **(b)** shows the distance measure L(t) versus the time from perturbation $t - t^{*}$ where t^{*} is the time of perturbation in our numerical simulation for one example parameter set. Before the perturbation (gray dotted line at $t - t^{*} = 0$) L(t) is identical to 0. L(0) shows how far the perturbed system altered from the underlying stable steady state. After perturbation, i.e. when $t - t^{*} > 0$, L(t) approaches 0 showing the system is Lyapunov stable.

Supplementary Table 1: Parameters used for numerical simulations of neural field model in the phase space analysis.

**Parameter**	**Value**	**Definition**
Δt	0.01	Time Step
T	150	Final Time
α_{EE}	$\in [0.25, 15]$	Diffusive strength
α_{EI}	$\in [0.25, 15]$	Diffusive strength
α_{IE}	$\in [0.25, 15]$	Diffusive strength
α_{II}	$\in [1, 45]$	Diffusive strength
σ^2_{EE}	$\in [250, 310]$	Diffusive speed
σ^2_{EI}	$\in [290, 370]$	Diffusive speed
σ^2_{IE}	$\in [290, 340]$	Diffusive speed
σ^2_{II}	$\in [260, 310]$	Diffusive speed
d_{E}	2	Excitatory decay rate
d_{I}	2	Inhibitory decay rate
F_c	2000	Use last F_c steps for Fourier Analysis
F_p	$\frac{100}{\Delta t}$	Maximum frequency plotted for Fourier Analysis

Supplementary Note 1: Neural Field Model and generalised Wilson-Cowan equations

Neural field models based on Wilson-Cowan equations are a variant of reaction-diffusion systems[3] originally introduced by Turing as a mathematical model for morphogenesis [4]. Based on the principle that mutual interactions between the elements of a system can result in self-organization and pattern formation, reaction-diffusion models have provided valuable insights into the mechanisms underlying the emergence of non-linear waves in several biological processes[5]. The interacting elements in a reaction-diffusion system can vary form discrete entities like molecules to circuits of cellular signals[5]. The interactions themselves can also be provided by a variety of mechanisms, e.g., a diffusion-like mechanism spreading the activity of interacting elements[4, 5], a series of direct cell-to-cell signals[6] or neuronal interactions[7]. When an asymmetry between the different spatial propagation of elements exists, such as the differences in diffusion rates of reactant chemicals[4], different spatial ranges of excitatory and inhibitory connections between neural populations[8] or different intra-cellular reaction speeds of the excitatory and inhibitory neural activity[6], the activity of the system can evolve spontaneously towards periodic patterns and self-organization occurs (Supplementary Fig. 7).

Here, we first describe a variant of the neural field model of cortical dynamics given by Wilson-Cowan equations[9] and then derive the extension of these equations to the 3D connectome model.

Let $E(x,t)$ and $I(x,t)$ correspond to the activity; i.e. local spatio-temporal averages of membrane-potentials, of the excitatory and inhibitory neurons at the cortical location $x \in \mathbb{R}^3$ at time t. Following the Wilson-Cowan equations[9, 8], time evolution of the excitatory and inhibitory neural firing rates satisfy the following non-linear differential equations[8]:

$$\tau_s \frac{\partial E(x,t)}{\partial t} = -d_{\mathsf{E}} E(x,t) + S(\alpha_{\mathsf{EE}} \mathcal{D}_{\mathsf{EE}}[E(x,t)] - \alpha_{\mathsf{IE}} \mathcal{D}_{\mathsf{IE}}[I(x,t)]) \tag{1}$$

$$\tau_s \frac{\partial I(x,t)}{\partial t} = -d_{\mathsf{I}} I(x,t) + S(\alpha_{\mathsf{EI}} \mathcal{D}_{\mathsf{EI}}[E(x,t)] - \alpha_{\mathsf{II}} \mathcal{D}_{\mathsf{II}}[I(x,t)]). \tag{2}$$

where $\mathcal{D}_{\mathsf{EE}}$, $\mathcal{D}_{\mathsf{IE}}$, $\mathcal{D}_{\mathsf{EI}}$ and $\mathcal{D}_{\mathsf{II}}$ denote the diffusion (spatial propagation) operators of excitatory (E) and inhibitory activities (I), each separately acting on excitatory and inhibitory populations with names (EE, IE) and (EI, II) respectively. Here S denotes the sigmoidal activation function (SAF),

$$S(r) = \frac{1}{1+e^{-r}}, \tag{3}$$

and τ_s is a characteristic time scale that speeds up or slows down the evolution of the system. In the rest of the supplementary material, without loss of generality, we will set $\tau_s = 1$ sec. for the simplicity of the presentation. However, it is important to note that the neural field model can be adapted to another frequency range of oscillations by changing the value of τ_s.

In continuous 2D domain, these diffusion operators can be replaced by integration against the diffusion (Gaussian) kernels $G(x,y,\sigma)$ with corresponding standard deviations $\sigma_{\{\mathsf{EE}\backslash\mathsf{IE}\backslash\mathsf{EI}\backslash\mathsf{II}\}}$ (Supplementary Fig. 7)[9, 8]:

$$\mathcal{D}_{\mathsf{EE}}[u(x,t)] = \int_\Omega G(x,y;\sigma_{\mathsf{EE}})u(x,t)dy, \tag{4}$$

$$\mathcal{D}_{\mathsf{IE}}[u(x,t)] = \int_\Omega G(x,y;\sigma_{\mathsf{IE}})u(x,t)dy, \tag{5}$$

$$\mathcal{D}_{\mathsf{EI}}[u(x,t)] = \int_\Omega G(x,y;\sigma_{\mathsf{EI}})u(x,t)dy, \tag{6}$$

$$\mathcal{D}_{\mathsf{II}}[u(x,t)] = \int_\Omega G(x,y;\sigma_{\mathsf{II}})u(x,t)dy, \tag{7}$$

where $\Omega \subset \mathbb{R}^3$, σ_{EE}, σ_{EI} denote the spatial ranges of excitatory activity acting on excitatory and inhibitory neural populations, respectively. Similarly, σ_{IE}, σ_{II} denote the spatial ranges of inhibitory activity acting on excitatory and inhibitory neural populations.

Supplementary Note 2: Diffusion on the human connectome

To compute the diffusion operators $\mathcal{D}_{\{\mathsf{EE}\backslash\mathsf{IE}\backslash\mathsf{EI}\backslash\mathsf{II}\}}$ on a 3D graph model of the human connectome, we draw on the evolution of the diffusion equation on the graph. To this end, we form the connectome graph and compute the symmetric graph Laplacian explained in Methods Section of the main article.

We now derive the diffusion equations on the graph that are analogous to the Gaussian diffusion kernels in the continuum. We do this by first considering the diffusive process in some small time Δt on vertex v_i. The explicit time discretisation of the diffusion equation

$$\frac{du(v_i,t)}{dt} = \Delta_{\mathcal{G}} u(v_i,t) \tag{8}$$

is

$$u(v_i, t+\Delta t) = u(v_i,t) + \Delta t \Delta_{\mathcal{G}} u(v_i,t) + o(\Delta t) \tag{9}$$

where Δt is small.

We expand $u(v_i,t)$ with respect to the orthogonal Laplacian eigenfunctions ψ_j (these eigenfunctions will also be used to conduct the linear stability analysis in Supplementary Notes 4 and 5):

$$\Delta_{\mathcal{G}}\psi_j(v_i) = \lambda_j \psi_j(v_i), \quad \forall v_i \in \mathcal{V} \tag{10}$$

with λ_j, $j \in \{1,...,n\}$ being the corresponding eigenvalues of $\Delta_{\mathcal{G}}$. Please note that the eigenvalues of the continuous Laplace operator are infinite and countable, whereas the eigenvalues of the graph Laplacian $\Delta_{\mathcal{G}}$ is limited by the number of nodes n of the graph $\mathcal{G}$. This expansion gives us:

$$u(v_i,t) = \sum_{j=1}^{n} \beta_j(t)\psi_j(v_i). \tag{11}$$

where $\beta(t)$ is some scalar function of time. Using Eq. (11) and negating the $o(\Delta t)$ term, we may rewrite Eq. (9) as

$$u(v_i, t+\Delta t) = (1+\Delta t \Delta_{\mathcal{G}}) \sum_{j=1}^{n} \beta_j(t)\psi_j(v_i) \tag{12}$$

$$= \sum_{j=1}^{n} \beta_j(t)(1+\Delta t \lambda_j)\psi_j(v_i). \tag{13}$$

We now observe how the diffusive process operates over the characteristic time τ, which corresponds to the Gaussion kernel $G(x,y;\sigma)$ with the standard deviation σ in 2D continuous setting. Note that $N=\tau/\Delta t$ denotes the number of discrete time steps and $\tau=\frac{\sigma^2}{2}$. To evaluate $u(v_i,t+\tau)$ we simply expand Eq. (13) giving:

$$\begin{aligned} u(v_i,t+\tau) &= \sum_{j=1}^{n}\beta_j(t+\tau-\Delta t)(1+\Delta t\lambda_j)\psi_j(v_i) && (14)\\ &= \sum_{j=1}^{n}\beta_j(t+\tau-2\Delta t)(1+\Delta t\lambda_j)^2\psi_j(v_i) && (15)\\ &\vdots \\ &= \sum_{j=1}^{n}\beta_j(t)(1+\Delta t\lambda_j)^N\psi_j(v_i). && (16)\end{aligned}$$

In the reaction-diffusion equations, we are interested in the diffusion process occurring between the time t and $t+\tau$, which denote the times of reactions in the discrete setting. In a purely diffusive process, one would consider the diffusion between the time $t_0=0$ and $t_0=\tau$ and thus the Eq. (16) would correspond to

$$u(v_i,\tau)=\sum_{j=1}^{n}\beta_j(0)(1+\Delta t\lambda_j)^N\psi_j(v_i). \qquad (17)$$

To see how this discrete approximation limits to the continuum Gaussian graph diffusion operator, $\Delta t\to 0$, we let $\Delta t=1/\eta$ and consider the limit of the Δt term in the right hand side of Eq. (17):

$$\begin{aligned} \lim_{\eta\to\infty}(1+\Delta t\lambda_j)^{\frac{t}{\Delta t}} &= \lim_{\eta\to\infty}(1+\frac{1}{\eta}\lambda_j)^{\eta t} && (18)\\ &= \left(\lim_{\eta\to\infty}(1+\frac{1}{\eta}\lambda_j)^{\eta}\right)^t && (19)\\ &= e^{\lambda_j t} && (20)\\ &= e^{\frac{\lambda_j\sigma^2}{2}}. && (21)\end{aligned}$$

We can then approximate the diffusion operators on the 3D graph with the exponential propagator as:

$$\mathcal{D}_{\mathsf{EE}}[u(v_i,t)]=\sum_{j=1}^{n}\beta_j(t)e^{\frac{\lambda_j\sigma^2}{2}}\psi_j(v_i), \qquad (22)$$

with corresponding definitions for (EE), (EI), (IE), (II). Please note that due to the particular graph Laplacian used in the simulations, (see Methods in the main article), all $\lambda_i\leq 0$. Thus, higher frequencies corresponding to larger negative eigenvalues get more attenuated/damped than lower frequencies, consistent with the continuous form of the diffusion process.

Supplementary Note 3. Relation of Wilson-Cowan equations to connectome harmonics

We use the neural field equation given in[9] as such equations can typically be derived from an underlying stochastic process and used in the network setting[10]. This approach allows for the derivation of master equations which yield physically consistent oscillatory behavior across the network[11]. Systems consisting

of a network of coupled oscillators are also effective models of macro-scale cortical dynamics which assume all nodes are natural oscillators[12, 13]. In contrast, reaction-diffusion dynamics can be derived from first principles without assuming oscillations. In such systems, the oscillatory behavior is an emergent phenomena.

To determine the necessary and sufficient conditions for the self-organization of spatial-temporal patterns, i.e., oscillatory networks, we will analyse first the stability of any homogeneous solution and then how small perturbations from a stable homogeneous solution evolve. This is a standard dynamical systems approach and the reader is advised to consult[14] for detailed explanation and analysis.

Let $\bar{E}$, $\bar{I}$ be a homogeneous state, i.e., $\bar{E}(v_i,t) = \bar{E}(v_j,t)$ for all v_i, v_j for any given t. In a homogeneous steady state, there is no net diffusion at any location. We consider the behavior of E through analysis of the temporal derivative $\frac{dE(v_i,t)}{dt}$ for all $v_i \in \mathcal{V}$. Specifically we substitute $\bar{E}(v_i,t)$ and $\bar{I}(v_i,t)$ into the set of master equations Eqs. (1) and (2) for all $v_i \in \mathcal{V}$;

$$\frac{d\bar{E}(v_i,t)}{dt} = -d_{\mathsf{E}}\bar{E}(v_i,t) + S(\alpha_{\mathsf{EE}}\mathcal{D}_{\mathsf{EE}}[\bar{E}(v_i,t)] - \alpha_{\mathsf{II}}\mathcal{D}_{\mathsf{IE}}[\bar{I}(v_i,t)]). \tag{23}$$

As $\bar{E}$ and $\bar{I}$ is a homogeneous solution, $\mathcal{D}_{\mathsf{EE}}[\bar{E}(v_i,t)] = \mathcal{D}_{\mathsf{IE}}[\bar{I}(v_i,t)] = 0$ and the right most term is simply

$$S(0) = \frac{1}{1+e^0} = \frac{1}{2}. \tag{24}$$

Thus

$$\frac{d\bar{E}(v_i,t)}{dt} = -d_{\mathsf{E}}\bar{E}(v_i,t) + \frac{1}{2} \tag{25}$$

$$> 0 \text{ if } \bar{E}(v_i,t) < \frac{1}{2d_{\mathsf{E}}}, \quad \text{or} < 0 \text{ if } \bar{E}(v_i,t) > \frac{1}{2d_{\mathsf{E}}}. \tag{26}$$

So we have positive growth for initial conditions below $\frac{1}{2d_{\mathsf{E}}}$ and negative growth for initial conditions above $\frac{1}{2d_{\mathsf{E}}}$. An identical approach may be used for $\bar{I}(v_i,t)$. This shows that any homogeneous state will converge onto the homogeneous steady state

$$E^0(v_i,t) = \frac{1}{2d_{\mathsf{E}}}, \quad I^0(v_i,t) = \frac{1}{2d_{\mathsf{I}}}, \quad \forall v_i. \tag{27}$$

To determine the stability of the state $E^0(v_i,t) = \frac{1}{2d_{\mathsf{E}}}$, $I^0(v_i,t) = \frac{1}{2d_{\mathsf{I}}}$ we consider a small, non-homogeneous perturbation, E^* and I^*, from $E^0(v_i,t), I^0(v_i,t)$;

$$E^*(v_i,0), I^*(v_i,0) > 0. \tag{28}$$

The homogeneous steady state $E^0(v_i,t), I^0(v_i,t)$ is unstable if

$$\frac{dE^*(v_i,t)}{dt} > 0 \text{ and } \frac{dI^*(v_i,t)}{dt} > 0 \text{ for } E^*(v_i,0), I^*(v_i,0) > 0 \tag{29}$$

and if

$$\frac{dE^*(v_i,t)}{dt} < 0 \text{ and } \frac{dI^*(v_i,t)}{dt} < 0 \text{ for } E^*(v_i,0), I^*(v_i,0) < 0. \tag{30}$$

The state of the system may be described as the sum of the perturbation E^* and the steady state E^0,

$$E(v_i,t) = E^0(v_i,t) + E^*(v_i,t). \tag{31}$$

The temporal behaviour is then given by

$$\begin{aligned}\frac{dE(v_i,t)}{dt} &= \frac{d(E^0(v_i,t)+E^*(v_i,t))}{dt}\\ &= -d_{\mathsf{E}}E^0(v_i,t)-d_{\mathsf{E}}E^*(v_i,t)\\ &\quad +S(\alpha_{\mathsf{EE}}\mathcal{D}_{\mathsf{EE}}[E^0(v_i,t)+E^*(v_i,t)]-\alpha_{\mathsf{II}}\mathcal{D}_{\mathsf{IE}}[I^0(v_i,t)+I^*(v_i,t)]).\end{aligned} \tag{32}$$

As $E^0(v_i,t)$ is a steady state $\frac{dE^0(v_i,t)}{dt}=0$. Thus we have the identification

$$\frac{dE(v_i,t)}{dt}=\frac{dE^*(v_i,t)}{dt}, \tag{33}$$

and Eq. (32) becomes:

$$\begin{aligned}\frac{dE^*(v_i,t)}{dt} &= -d_{\mathsf{E}}E^0(v_i,t)-d_{\mathsf{E}}E^*(v_i,t)\\ &\quad +S(\alpha_{\mathsf{EE}}\mathcal{D}_{\mathsf{EE}}[E^0(v_i,t)+E^*(v_i,t)]-\alpha_{\mathsf{II}}\mathcal{D}_{\mathsf{IE}}[I^0(v_i,t)+I^*(v_i,t)])\\ &= -\frac{1}{2}-d_{\mathsf{E}}E^*(v_i,t)+S(\alpha_{\mathsf{EE}}\mathcal{D}_{\mathsf{EE}}[E^*(v_i,t)]-\alpha_{\mathsf{II}}\mathcal{D}_{\mathsf{IE}}[I^*(v_i,t)])\end{aligned} \tag{34}$$

as $E^0(v_i,t)=\frac{1}{2d_{\mathsf{E}}}$, from Eq. (27), and

$$\mathcal{D}_{\mathsf{EE}}[E^0(v_i,t)+E^*(v_i,t)]=\mathcal{D}_{\mathsf{EE}}[E^0(v_i,t)]+\mathcal{D}_{\mathsf{EE}}[E^*(v_i,t)]=\mathcal{D}_{\mathsf{EE}}[E^*(v_i,t)] \tag{35}$$

as $\mathcal{D}_{\mathsf{EE}}[E^0(v_i,t)]=0$.

Then, by taking the Taylor series expansion of $S(c)$ about 0, i.e., $S(c)=\frac{1}{2}+\frac{c}{4}+\mathcal{O}(c)$, we arrive at

$$\frac{dE^*(v_i,t)}{dt} \approx -\frac{1}{2}-d_{\mathsf{E}}E^*(v_i,t)+\frac{1}{2}+\frac{\alpha_{\mathsf{EE}}\mathcal{D}_{\mathsf{EE}}[E^*(v_i,t)]-\alpha_{\mathsf{II}}\mathcal{D}_{\mathsf{IE}}[I^*(v_i,t)]}{4} \tag{36}$$

$$= -d_{\mathsf{E}}E^*(v_i,t)+\frac{\alpha_{\mathsf{EE}}\mathcal{D}_{\mathsf{EE}}[E^*(v_i,t)]-\alpha_{\mathsf{IE}}\mathcal{D}_{\mathsf{IE}}[I^*(v_i,t)]}{4}. \tag{37}$$

Similarly for I, we obtain

$$\frac{dI^*(v_i,t)}{dt}\approx -d_{\mathsf{I}}I^*(v_i,t)+\frac{\alpha_{\mathsf{EI}}\mathcal{D}_{\mathsf{EI}}[E^*(v_i,t)]-\alpha_{\mathsf{II}}\mathcal{D}_{\mathsf{II}}[I^*(v_i,t)]}{4}. \tag{38}$$

The perturbations $E^*(v_i,t)$ and $I^*(v_i,t)$ are defined over the basis of Laplacian eigenfunctions:

$$E^*(v_i,t)=\sum_{j=1}^{N}\beta_j^E(t)\psi_j(v_i) \qquad I^*(v_i,t)=\sum_{j=1}^{N}\beta_j^I(t)\psi_j(v_i). \tag{39}$$

It is important to note that these eigenfunctions ψ_j are the connectome harmonics discussed in the main article. Eq. (39) states that any excitatory or inhibitory state can be reconstructed through the a finite number of eigenfunctions. In Fig. 2 and Supplementary Figs. 2-5, we showed that the oscillatory functional networks of the human brain in resting state directly correspond to a small number of connectome harmonics.

Supplementary Note 4: Necessary conditions for pattern formation

We now analyse the behaviour of $\beta_j^{E/I}(t)$ to determine which, if any, eigenmodes are unstable and will dominate to give patterns. We substitute this perturbation into the master equations Eqs. (37) and (38):

$$\begin{aligned}
\frac{\partial}{\partial t}\sum_{j=1}^{N}\beta_j^E(t)\psi_j(v_i) &= -d_{\mathsf{E}}\sum_{j=1}^{N}\beta_j^E(t)\psi_j(v_i) \\
&+ \frac{\alpha_{\mathsf{EE}}D_{\mathsf{EE}}\left[\sum_{j=1}^{N}\beta_j^E(t)\psi_j(v_i)\right] - \alpha_{\mathsf{IE}}D_{\mathsf{IE}}\left[\sum_{j=1}^{N}\beta_j^I(t)\psi_j(v_i)\right]}{4}, \qquad (40)\\
\frac{\partial}{\partial t}\sum_{j=1}^{N}\beta_j^I(t)\psi_j(v_i) &= -d_{\mathsf{I}}\sum_{j=1}^{N}\beta_j^I(t)\psi_j(v_i) \\
&+ \frac{\alpha_{\mathsf{EI}}D_{\mathsf{EI}}\left[\sum_{j=1}^{N}\beta_j^E(t)\psi_j(v_i)\right] - \alpha_{\mathsf{II}}D_{\mathsf{II}}\left[\sum_{j=1}^{N}\beta_j^I(t)\psi_j(v_i)\right]}{4}. \qquad (41)
\end{aligned}$$

As the eigenfunctions are orthogonal (i.e. the matrix of all ψ is linearly independent) we can look at each eigenfunction individually. To simplify our analysis, we replace the diffusion term with its propagator, Eq. (22). The time evolution of each eigenfunction is then given by the linearised equation

$$\begin{aligned}
\frac{\partial\beta_j^E(t)\psi_j(v_i)}{\partial t} &\approx -d_{\mathsf{E}}\beta_j^E(t)\psi_j(v_i) + \frac{\alpha_{\mathsf{EE}}\beta_j^E(t)\exp\left(\lambda_j\frac{\sigma_{\mathsf{EE}}^2}{2}\right)\psi_j(v_i) - \alpha_{\mathsf{IE}}\beta_j^I(t)\exp\left(\lambda_j\frac{\sigma_{\mathsf{IE}}^2}{2}\right)\psi_j(v_i)}{4},\\
\frac{\partial\beta_j^I(t)\psi_j(v_i)}{\partial t} &\approx -d_{\mathsf{I}}\beta_j^I(t)\psi_j(v_i) + \frac{\alpha_{\mathsf{EI}}\beta_j^E(t)\exp\left(\lambda_j\frac{\sigma_{\mathsf{EI}}^2}{2}\right)\psi_j(v_i) - \alpha_{\mathsf{II}}\beta_j^I(t)\exp\left(\lambda_j\frac{\sigma_{\mathsf{II}}^2}{2}\right)\psi_j(v_i)}{4}. \qquad (42)
\end{aligned}$$

We can rewrite the system of equations defined in Eqs. (42) as

$$\frac{\partial}{\partial t}\begin{pmatrix}\beta_j^E(t)\\ \beta_j^I(t)\end{pmatrix} = \begin{pmatrix} -d_{\mathsf{E}} + \frac{\alpha_{\mathsf{EE}}e^{\left(\lambda_j\frac{\sigma_{\mathsf{EE}}^2}{2}\right)}}{4} & -\frac{\alpha_{\mathsf{IE}}e^{\left(\lambda_j\frac{\sigma_{\mathsf{IE}}^2}{2}\right)}}{4}\\ \frac{\alpha_{\mathsf{EI}}e^{\left(\lambda_j\frac{\sigma_{\mathsf{EI}}^2}{2}\right)}}{4} & -d_{\mathsf{I}} - \frac{\alpha_{\mathsf{II}}e^{\left(\lambda_j\frac{\sigma_{\mathsf{II}}^2}{2}\right)}}{4}\end{pmatrix}\begin{pmatrix}\beta_j^E(t)\\ \beta_j^I(t)\end{pmatrix} \qquad (43)$$

(44)

or equivalently in the matrix format given by

$$\frac{\partial}{\partial t}\bar{\beta}_j(t) = \mathbf{H_j}\bar{\beta}_j(t). \qquad (45)$$

To obtain a patterned system, i.e., different vertices have different concentrations across the graph, we require that any perturbation away from the homogeneous steady state to grow. Formally we require

the determinant of $\mathbf{H_j} > 0$;

$$
\begin{aligned}
\det(\mathbf{H_j}) &= \left(-d_{\mathsf{E}} + \frac{\alpha_{\mathsf{EE}} e^{\left(\lambda_j \frac{\sigma_{\mathsf{EE}}^2}{2}\right)}}{4}\right)\left(-d_{\mathsf{I}} - \frac{\alpha_{\mathsf{II}} e^{\left(\lambda_j \frac{\sigma_{\mathsf{II}}^2}{2}\right)}}{4}\right) + \frac{\alpha_{\mathsf{IE}} e^{\left(\lambda_j \frac{\sigma_{\mathsf{IE}}^2}{2}\right)}}{4} \frac{\alpha_{\mathsf{EI}} e^{\left(\lambda_j \frac{\sigma_{\mathsf{EI}}^2}{2}\right)}}{4} \\
&= d_{\mathsf{E}} d_{\mathsf{I}} - d_{\mathsf{I}} \frac{\alpha_{\mathsf{EE}} e^{\left(\lambda_j \frac{\sigma_{\mathsf{EE}}^2}{2}\right)}}{4} + d_{\mathsf{E}} \frac{\alpha_{\mathsf{II}} e^{\left(\lambda_j \frac{\sigma_{\mathsf{II}}^2}{2}\right)}}{4} - \frac{\alpha_{\mathsf{EE}} \alpha_{\mathsf{II}} e^{\left(\lambda_j \frac{\sigma_{\mathsf{EE}}^2 + \sigma_{\mathsf{II}}^2}{2}\right)}}{16} + \frac{\alpha_{\mathsf{IE}} \alpha_{\mathsf{EI}} e^{\left(\lambda_j \frac{\sigma_{\mathsf{IE}}^2 + \sigma_{\mathsf{EI}}^2}{2}\right)}}{16} \\
&> 0, \qquad (46)
\end{aligned}
$$

or equivalently if

$$
16 d_{\mathsf{E}} d_{\mathsf{I}} - 4 d_{\mathsf{I}} \alpha_{\mathsf{EE}} e^{\left(\lambda_j \frac{\sigma_{\mathsf{EE}}^2}{2}\right)} + 4 d_{\mathsf{E}} \alpha_{\mathsf{II}} e^{\left(\lambda_j \frac{\sigma_{\mathsf{II}}^2}{2}\right)} - \alpha_{\mathsf{EE}} \alpha_{\mathsf{II}} e^{\left(\lambda_j \frac{\sigma_{\mathsf{EE}}^2 + \sigma_{\mathsf{II}}^2}{2}\right)} + \alpha_{\mathsf{IE}} \alpha_{\mathsf{EI}} e^{\left(\lambda_j \frac{\sigma_{\mathsf{IE}}^2 + \sigma_{\mathsf{EI}}^2}{2}\right)} > 0. \qquad (47)
$$

We note that this is a necessary but not sufficient condition for obtaining patterned systems.

Supplementary Note 5: Necessary condition for oscillations

To obtain an oscillatory steady state, we require the eigenvalues of $\mathbf{H_j}$ to have imaginary components or equivalently, $\mathrm{tr}(\mathbf{H_j})^2 - 4\det(\mathbf{H_j}) < 0$. The point in phase space where the eigenvalues become imaginary is known as a Hopf bifurcation. Using the definition of $\mathbf{H_j}$ in Eq. (45); the necessary condition for oscillations is:

$$
\begin{aligned}
\mathrm{tr}(\mathbf{H_j})^2 - 4\det(\mathbf{H_j}) &= \left(-d_{\mathsf{E}} - d_{\mathsf{I}} + \frac{\alpha_{\mathsf{EE}} e^{\left(\frac{\sigma_{\mathsf{EE}}^2}{2}\lambda_j\right)}}{4} - \frac{\alpha_{\mathsf{II}} e^{\left(\frac{\sigma_{\mathsf{II}}^2}{2}\lambda_j\right)}}{4}\right)^2 \\
&- 4\left(d_{\mathsf{E}} d_{\mathsf{I}} - d_{\mathsf{I}} \frac{\alpha_{\mathsf{EE}} e^{\left(\lambda_j \frac{\sigma_{\mathsf{EE}}^2}{2}\right)}}{4} + d_{\mathsf{E}} \frac{\alpha_{\mathsf{II}} e^{\left(\lambda_j \frac{\sigma_{\mathsf{II}}^2}{2}\right)}}{4} - \frac{\alpha_{\mathsf{EE}} \alpha_{\mathsf{II}} e^{\left(\lambda_j \frac{\sigma_{\mathsf{EE}}^2 + \sigma_{\mathsf{II}}^2}{2}\right)}}{16} + \frac{\alpha_{\mathsf{IE}} \alpha_{\mathsf{EI}} e^{\left(\lambda_j \frac{\sigma_{\mathsf{IE}}^2 + \sigma_{\mathsf{EI}}^2}{2}\right)}}{16}\right) \\
&= \left(-d_{\mathsf{E}} + d_{\mathsf{I}} + \frac{\alpha_{\mathsf{EE}} e^{\left(\frac{\sigma_{\mathsf{EE}}^2}{2}\lambda_j\right)}}{4} + \frac{\alpha_{\mathsf{II}} e^{\left(\lambda_j \frac{\sigma_{\mathsf{II}}^2}{2}\right)}}{4}\right)^2 - \frac{\alpha_{\mathsf{EI}} \alpha_{\mathsf{IE}} e^{\left(\lambda_j \frac{\sigma_{\mathsf{IE}}^2 + \sigma_{\mathsf{EI}}^2}{2}\right)}}{4} \qquad (48) \\
&< 0. \qquad (49)
\end{aligned}
$$

It is very important to note that this condition is a necessary but not a sufficient condition for oscillations. This is due to the different behavior of sub- and super-critical Hopf regimes. A Hopf regime permits at least two points in phase space, an oscillatory one and a stationary one. One of these points is stable whilst the other is unstable. A point in phase space is stable if different perturbations in the phase space converge in some finite period of time. The unstable point is a steady state however any perturbation will push it out of this state.

An illustrative example of the different types of steady state can be observed by a rigid pendulum which will converge to its stable state, with all weight at the lowest possible point, after some finite time. This is the stable point of the system. The unstable point in this system is to perfectly balance the pendulum at the top of its cycle, where the pendulum is at its highest point. The pendulum may stay there if not pushed. However any small perturbation, i.e. a breath of wind, will push it out of this steady state. It is impossible for the pendulum to stay there for a long time. Hence such a point is known as unstable (see Supplementary Fig. 10).

If the stable point is oscillatory, the set of parameters can be described as belonging to a super-critical Hopf regime. If the stable point in phase space is stationary, then any perturbation will converge to this stationary steady state in some finite time. In this case, the parameter set is described as belonging to a sub-critical Hopf regime. A stable oscillatory state can be observed through the oscillatory behaviour of the state space variables, $E(v_i, t)$ and $I(v_i, t)$, in the long time limit.

Due to the complexity and high dimensionality of the system we do not have an algebraic form to determine which parameters allow for super-critical Hopf regimes. We can only numerically test different parameters sets satisfying Eq. (49) to obtain oscillatory behavior. As shown in Fig. 3 in the main article and Supplementary Fig. 8, we observe a direct relationship between the entire Hopf regime and region of observed oscillations.

Supplementary Methods

Comparison to resting state networks:

We quantitatively evaluate of the agreement between each connectome harmonic ψ_j and each of the 7 resting state networks $\mathrm{f}_{\{\mathrm{RSN}\}}$ with RSN $\in$ {default mode network, control network, dorsal attention network, ventral attention network, visual network, limbic network, somato-motor network} using two different measures: mutual information and F-measure.

An important challenge for the quantitative comparison between the connectome harmonics and the resting state networks is introduced by the fact that the reference resting state networks are labeled as binary patterns, given by indicator functions $\mathrm{f}_{\{\mathrm{RSN}\}} : \mathcal{V} \to \{0, 1\}$ with

$$\mathrm{f}_{\{\mathrm{RSN}\}}(v_i) = \begin{cases} 1 & \text{if } v_i \in \mathrm{RSN} \\ 0 & \text{otherwise,} \end{cases} \tag{50}$$

whereas the connectome harmonics ψ_j are smooth functions $\psi_j : \mathcal{V} \to [-1, 1]$. This induces a non-linear relation between the resting state networks and the connectome harmonics. To address this challenge, we utilize two different measures.

Mutual information:

First, we evaluate the similarity between each connectome harmonic and each of the 7 resting state networks using mutual information, an information theoretical measure quantifying the non-linear dependence of two random variables, computed as

$$\mathrm{MI}(\psi_j, \mathrm{f}_{\{\mathrm{RSN}\}}) = \sum_{v_i \in \mathcal{V}} p\left(\psi_j(v_i), \mathrm{f}_{\{\mathrm{RSN}\}}(v_i)\right) \log \frac{p\left(\psi_j(v_i), f_{\mathrm{RSN}}(v_i)\right)}{p(\psi_j(v_i)) \cdot p(f_{\mathrm{RSN}}(v_i))} \quad , \tag{51}$$

where $p\left(\psi_j(v_i), \mathrm{f}_{\{\mathrm{RSN}\}}(v_i)\right)$ denotes the joint probability distributions of the random variables ψ_j and f_{RSN} and $p(\psi_j)$ and $p(f_{\mathrm{RSN}})$ denote the marginal entropies of ψ_j and f_{RSN}, respectively.

Mutual information measures the mutual dependence of the two random variables ($\mathrm{MI}(X, Y) = 0$ if X and Y are independent, whereas the maximum value of $\mathrm{MI}(X, Y)$ depends on the individual entropy values of X and Y) and offers the advantages of capturing non-linear relations and being model independent (no parametric model is assumed for X or Y). Thus, mutual information provides a suitable measure to evaluate the similarity of an indicator and a smooth function, as in the case of resting state networks and connectome harmonics.

F-measure:

In the second evaluation, we turn the smooth connectome harmonics into binary indicator functions defined as:

$$\Psi_{\mathrm{j}}(v_i) = \begin{cases} 1 & \text{if } \psi_j > 0 \\ 0 & \text{otherwise,} \end{cases} \tag{52}$$

and utilize the information retrieval measure, called F-measure, to simultaneously quantify the recall and precision of the binarized connectome harmonics' prediction of the resting state networks. To this end, for each comparison (between one binarized connectome harmonic pattern Ψ_{j} and one resting state network $\mathrm{f}_{\{\mathrm{RSN}\}}$) we compute the confusion matrix for each vertex $v_i \in \mathcal{V}$ as follows:

**Resting state network**	**Connectome harmonic value** $\Psi_{\mathrm{j}}(v_i) = 1$	**Connectome harmonic value** $\Psi_{\mathrm{j}}(v_i) = 0$
$\mathrm{f}_{\{\mathrm{RSN}\}}(v_i) = 1$	True Positive	False Negative
$\mathrm{f}_{\{\mathrm{RSN}\}}(v_i) = 0$	False Positive	True Negative

Then we compute the recall (R), precision (P) and F-measure (FM) - a harmonic mean between the precision and recall - values for each comparison as:

$$\mathrm{R}(\Psi_{\mathrm{j}}, \mathrm{f}_{\{\mathrm{RSN}\}}) = \frac{\left|\{v_i | \Psi_{\mathrm{j}}(v_i) = 1 \wedge \mathrm{f}_{\{\mathrm{RSN}\}}(v_i) = 1\}\right|}{\left|\{v_i | \Psi_{\mathrm{j}}(v_i) = 1 \wedge \mathrm{f}_{\{\mathrm{RSN}\}}(v_i) = 1\}\right| + \left|\{v_i | \Psi_{\mathrm{j}}(v_i) = 0 \wedge \mathrm{f}_{\{\mathrm{RSN}\}}(v_i) = 1\}\right|} \quad ,$$

$$\mathrm{P}(\Psi_{\mathrm{j}}, \mathrm{f}_{\{\mathrm{RSN}\}}) = \frac{\left|\{v_i | \Psi_{\mathrm{j}}(v_i) = 1 \wedge \mathrm{f}_{\{\mathrm{RSN}\}}(v_i) = 1\}\right|}{\left|\{v_i | \Psi_{\mathrm{j}}(v_i) = 1 \wedge \mathrm{f}_{\{\mathrm{RSN}\}}(v_i) = 1\}\right| + \left|\{v_i | \Psi_{\mathrm{j}}(v_i) = 1 \wedge \mathrm{f}_{\{\mathrm{RSN}\}}(v_i) = 0\}\right|} \quad ,$$

$$\mathrm{FM}(\Psi_{\mathrm{j}}, \mathrm{f}_{\{\mathrm{RSN}\}}) = \frac{2 \cdot \mathrm{R}(\Psi_{\mathrm{j}}, \mathrm{f}_{\{\mathrm{RSN}\}}) \cdot \mathrm{P}(\Psi_{\mathrm{j}}, \mathrm{f}_{\{\mathrm{RSN}\}})}{\mathrm{R}(\Psi_{\mathrm{j}}, \mathrm{f}_{\{\mathrm{RSN}\}}) + \mathrm{P}(\Psi_{\mathrm{j}}, \mathrm{f}_{\{\mathrm{RSN}\}})} \quad .$$

Thus the recall $\mathrm{R}(\Psi_{\mathrm{j}}, \mathrm{f}_{\{\mathrm{RSN}\}})$ and precision $\mathrm{P}(\Psi_{\mathrm{j}}, \mathrm{f}_{\{\mathrm{RSN}\}})$ values correspond to

$$\text{Recall} = \frac{\text{number of true positives}}{\text{number of true positives} + \text{number of false negatives}}$$

and

$$\text{Precision} = \frac{\text{number of true positives}}{\text{number of true positives} + \text{number of false positives}}$$

respectively and the F-measure approaches 1 when both, recall and precision of the prediction are optimized simultaneously.

Monte-Carlo simulations:

To determine whether the measured mutual information and F-measure values of the connectome harmonics for predicting the resting state networks were significantly different from the harmonics (Laplacian eigenfunctions) of a cortical network model with random white matter connections, we performed Monte Carlo simulations[2, 15] (2000 simulations per subject). Monte Carlo simulations base on the nonparametric randomization test and assume the independence of the observations, which is fulfilled in our data due to the independence of individual subjects and of the Laplacian eigenfunctions.

For each subject, we replaced the total number of long-range connections determined by the DTI fiber tractography by the same number of random connections, while preserving the local connections determined by the shape of the cortical surface of that subject. This results in a cortical network model that preserves the topology of the subjects cortical surface and thus the local connections, while the long-range connections are fully randomized. The preservation of the cortical anatomy enables the vertex-to-vertex comparison of the harmonics of the randomized cortical network model to the reference resting state networks. For each subject's simulation we estimated the harmonics of the randomized cortical network model following the same methodology for computing the connectome harmonics. We then computed the mutual information and the F-measure values for randomized harmonics and each resting state network, as described above. We evaluated the mutual information values of connectome harmonics and randomized harmonics for individual subjects as well as for mutual information and F-measure values averaged across all 10 subjects.

For individual subject analysis, we computed the p-values for the null hypothesis that the randomized harmonics predict the resting state networks with the same accuracy (measured in terms of mutual information and F-measure) for each subject separately and applied the false discovery rate correction to correct for multiple comparisons[2, 15].

For the group analysis, we averaged the mutual information and F-measure values of connectome harmonics across 10 subjects and compare to those of the randomized harmonics again averaged across 10 subjects. To form a group averaged simulation, we randomly choose one of 2000 randomized harmonics set for each subject and average the mutual information and F-measure values across all subjects. We performed a total number of 500.000 group-averaged simulations and computed the p-values after applying the false discovery rate correction to correct for multiple comparisons[2, 15].

Reconstruction error:

The orthogonality of the connectome harmonics implies that a linear combination of these eigenfunctions can be used as a function basis to represent any spatial pattern of neural activity. Furthermore, using the connectome harmonics - eigenvectors of the connectome Laplacian - as a function basis provides a natural extension of the Fourier transform. The classical (1D) Fourier transform decomposes of a signal into a linear combination of the eigenfunctions of the Laplace operator on the interval $[0, 2\pi]$ with cyclic boundary conditions, i.e., Laplacian applied to a circle[16].

To analyze the spatial frequency-content of the resting state networks, we performed a spectral transform to the connectome harmonic basis $\Psi = \{\psi_j\}_{j=1}^n$ and computed the normalized reconstruction error[17] for the spatial patterns of each individual resting state network as:

$$\epsilon_R(\mathrm{f}_{\{\mathrm{RSN}\}}) = \sqrt{\frac{\sum_{v_i \in \mathcal{V}} \left| \mathrm{f}_{\{\mathrm{RSN}\}}(v_i) - \mathrm{g}_{\{\mathrm{RSN}\}}(v_i) \right|^2}{\sum_{v_i \in \mathcal{V}} \left| \mathrm{f}_{\{\mathrm{RSN}\}}(v_i) \right|^2}}, \tag{53}$$

where $\mathrm{g}_{\{\mathrm{RSN}\}}$ denotes the RSN pattern reconstructed from the connectome harmonic basis $\Psi = \{\psi_j\}_{j=1}^n$:

$$\mathrm{g}_{\{\mathrm{RSN}\}} = \sum_{j=1}^{m} \left\langle \mathrm{f}_{\{\mathrm{RSN}\}}, \psi_j \right\rangle \psi_j. \tag{54}$$

Although the binary nature of the reference networks theoretically necessitates the use of the whole spectrum eigenmodes ($m = n$) for reconstruction - the same way that a square wave can only be reconstructed using the sine waves with infinite many frequencies, sharp decreases in the reconstruction errors are observed within 0.1% of the spectrum (low frequency range) (Fig. 2d, main article). The steepest decrease of the default mode network's reconstruction error occurred for the frequency band that also showed significant similarity and predictive power in mutual information and F-measure values, respectively (highlighted by the red column in Fig. 2d main article) while for the visual, somato-motor and limbic networks the decrease of the reconstruction error remained large but constant within 0.1% of the spectrum (Fig. 2d, main article). Slower convergence was observed for the reconstruction errors of higher cognitive networks within the range of 1.2% of the spectrum (low frequency range) suggesting the reliance of these networks on a broader range of the frequencies (Fig. 2d, main article). These results confirm our previous findings while providing a novel analytical language of cortical activity based on the classical Fourier transform, that can be utilised to quantify any activity pattern including task-based event related designs.

Phase space analysis:

To analyse the temporal behaviour of the system described in Eqs. (1) and (2), we numerically solve the master equation on a the graph representation of the human connectome. We use a simple explicit numerical scheme with step size Δt and solved for times $t \in [0, T]$.

The Maximal Lyapunov exponent (MLE) is typically used to check for numerical convergence. This value quantifies the rate of separation of close trajectories. A negative exponent means that two separate trajectories will converge whilst a positive exponent means that they diverge. We were not able to obtain the MLE analytically due the high dimensionality of the system. Hence we tested the convergence of different trajectories numerically. If the trajectories diverge, the system is either chaotic and/or the numerical method is unstable. If they converge, the system is non-chaotic and the numerical method is stable. We tested our neural field model for different parameter regimes by randomly perturbing the solution at a long time point and observing the resultant trajectory. Comparison of the perturbed trajectories to the original trajectory showed their convergence for $\Delta t = 0.01$ and also further confirmed for $\Delta t < 0.01$.

Fourier analysis:

After obtaining $T/\Delta t + 1$ temporal values for each node in the network, we perform a discrete Fourier transform on last F_c values for each node given in the matrix E. We obtain

$$\mathtt{F_E} = \mathtt{fft}(\mathtt{E}(:, \mathtt{end} - \mathtt{F_c} : \mathtt{end})) \tag{55}$$

where $\mathtt{F_E}$ is the power spectrum and the `fft` function in `Matlab` was used to perform the discrete Fourier transform. Only these last values are used as the system was found to be in some stable state by that time. Whether this stable state is oscillatory or stationary is determined by the parameter set.

The power spectrum was then averaged across all nodes, i.e.,

$$\mathtt{mean\_E} = \mathtt{mean}(\mathtt{abs}(\mathtt{F_E}(:, 2 : \mathtt{Fp} * \Delta\mathtt{t})), 1). \tag{56}$$

The first index is not considered as that corresponds to the stationary steady state.

This average power spectrum was plotted for varying values of the diffusion parameters $\alpha_{\{\mathsf{EE,IE,EI,II}\}}$ and $\sigma^2_{\{\mathsf{EE,IE,EI,II}\}}$ (see Fig. 3 in the main article and Supplementary Fig. 8). This was compared to regions where Eq. (49), the necessary condition for oscillation, was satisfied for varying values of λ, the eigenvalue of the Laplacian of the system.

Supplementary Fig. 8 shows how variations in α affect the necessary condition for oscillations obtained analytically (Eq. (49)) and the frequency of temporal oscillations in numerical solutions of the neural field equations (Eqs. (1), (2)) on the human connectome as discussed above. The red regions in Fig. 8A correspond to the diffusion parameters in the phase space that algebraically satisfy the necessary condition for oscillations, i.e., the critical Hopf regime, plotted as a function of the analysed diffusion parameter and the eigenvalue of the connectome harmonic. Connectome harmonics within the red region can be activated by the neural field model for the given parameter set (for each plot, α_{EE}, α_{IE}, α_{EI}, α_{II} are varied individually while fixing the remaining parameters to the following default values $d_{\mathsf{E}} = 2$, $d_{\mathsf{I}} = 2$, $\sigma^2_{\mathsf{EE}} = 6$, $\sigma^2_{\mathsf{IE}} = 10$, $\sigma^2_{\mathsf{EI}} = 10$, $\sigma^2_{\mathsf{II}} = 30$). Numerical solutions obtained by the Fourier frequency spectrum are shown in Supplementary Fig. 8b. We observe regions where temporal oscillations with certain dominant frequency (red) and its higher harmonics (yellow) are present, i.e., the region satisfies the super-critical Hopf regime, as well as where no oscillations are present (dark blue), i.e., the regions which may satisfy the sub-critical Hopf regime but do not lead to stable oscillatory behaviour. However, unlike variations of the diffusion speed, given by the $\sigma_{\{\mathsf{EE},\mathsf{IE},\mathsf{EI},\mathsf{II}\}}$ values in the neural field model (see Fig. 3 in the main article), we did not observe significant change in the dominant frequency of temporal oscillations for variations of $\alpha_{\{\mathsf{EE},\mathsf{IE},\mathsf{EI},\mathsf{II}\}}$ in the analysed parameter range.

Correlation analysis of neural field patterns:

For the analyzed range of the parameter space, we observed a decrease in the frequency of coherent oscillations when inhibitory activity is increased, modeled by faster diffusion of inhibition in the neural field model. In contrast, the dominant frequency increased with decreasing inhibitory or increasing excitatory activity (see Fig. 3b in the main article). This relationship between the frequency of temporal oscillations and the excitation-inhibition balance shows remarkable overlap with the neurophysiological changes observed during the loss and recovery of consciousness.

Neurophysiological evidence suggests that drug- or sleep-induced loss of consciousness is associated with increasing inhibitory or decreasing excitatory activity, which is accompanied by a transition from the low amplitude, high-frequency patterns to low frequency coherent oscillations in cortical activity[18, 19]. Recent work also shows changes in the spatial patterns of correlated oscillations, in particular gradual decoupling between the posterior and anterior midline nodes of the default mode network during loss of consciousness[20, 21].

To asses whether similar spatial changes occur in the correlation patterns of the neural field model, we performed seed-based correlation analysis on the numerical simulations for four different seed locations selected from the medial prefrontal cortex (MPC), posterior cingulate cortex (PCC), lateral parietal (LP) area, and middle temporal (MT) area. We observed spatial changes in the correlation patterns for decreasing excitatory and increasing inhibitory activity (Supplementary Fig. 9). In paricular, we found a decoupling between the anterior and posterior cortices for increased inhibitory and decreased excitatory activity (Supplementary Fig. 9d) which was also accompanied by slow oscillations in cortical activity (Fig. 3, main article, Supplementary Movies 1-4). This particular relation between the spatio-temporal frequency of neural field patterns and the excitation-inhibition balance remarkably overlaps with the neurophysiological changes observed during the loss and recovery of consciousness.

Testing the robusness of the neural field model to perturbations:

We tested the robustness of the neural field model in face of perturbations through Lyapunov's first method[22]. This method involves perturbing the system at some time t^* and observing whether the perturbed system converges to the original system. If this is the case, the system may be called Lyapunov stable.

As an analogy, one can consider the case of a simple rigid pendulum. To determine if the state of weight at the bottom is stable, we may move the weight from this state and observe the behaviour. It

is clear that the weight goes back to the bottom. Thus, the state where the weight is at the bottom is stable. If the weight is at the top, any small perturbation will push it away from that state and it will settle at the bottom after some time changing the (unstable) state of the system Supplementary Fig. 10a.

As the neural field model is oscillatory for the parameter sets we are concerned with, we must define a measure which determines the distance between two states. We first perturb the system separately 10 times by white noise at time t^*. We then take the absolute value of the difference at each node at each time step and taking the maximum difference. This is known as a L-infinity norm and is commonly used in stability analysis as it looks at the worst case rather than an average case. At each time step we compare all differences across the 10 perturbed systems and again take the worst case. This quantity is defined to be $\mathrm{L}(t)$. We plot the distance measure $\mathrm{L}(t)$ over time and observe whether it goes to 0 or some very small value. In Fig 10b we see that $\mathrm{L}(t)$ goes to some very small value and is bounded demonstrating that the system is Lyapunov stable.

Supplementary References

[1] Thomas Yeo, B. T. *et al.* The organization of the human cerebral cortex estimated by intrinsic functional connectivity. *Journal of Neurophysiology* **106**, 1125–1165 (2011).

[2] Benjamini, Y. & Hochberg, Y. Controlling the false discovery rate: a practical and powerful approach to multiple testing. *Journal of the Royal Statistical Society. Series B (Methodological)* 289–300 (1995).

[3] Bressloff, P. C. Spatiotemporal dynamics of continuum neural fields. *Journal of Physics A: Mathematical and Theoretical* **45**, 033001 (2012).

[4] Turing, A. M. The chemical basis of morphogenesis. *Philos. Trans. R. Soc. Lond. B Biol. Sci.* **237**, 37–72 (1952).

[5] Kondo, S. & Miura, T. Reaction-diffusion model as a framework for understanding biological pattern formation. *Science* **329**, 1616–1620 (2010).

[6] Rauch, E. M. & Millonas, M. M. The role of trans-membrane signal transduction in turing-type cellular pattern formation. *Journal of theoretical biology* **226**, 401–407 (2004).

[7] Swindale, N. A model for the formation of ocular dominance stripes. *Proceedings of the Royal Society of London. Series B. Biological Sciences* **208**, 243–264 (1980).

[8] Ermentrout, G. & Cowan, J. D. Temporal oscillations in neuronal nets. *Journal of mathematical biology* **7**, 265–280 (1979).

[9] Wilson, H. R. & Cowan, J. D. A mathematical theory of the functional dynamics of cortical and thalamic nervous tissue. *Kybernetik* **13**, 55–80 (1973).

[10] Angstmann, C. N., Donnelly, I. C. & Henry, B. I. Pattern formation on networks with reactions: A continuous-time random-walk approach. *Physical Review E* **87**, 032804 (2013).

[11] Hata, S., Nakao, H. & Mikhailov, A. S. Dispersal-induced destabilization of metapopulations and oscillatory turing patterns in ecological networks. *Scientific reports* **4** (2014).

[12] Kuramoto, Y. *Chemical oscillations, waves, and turbulence* (Courier Dover Publications, 2003).

[13] Deco, G., Jirsa, V. K. & McIntosh, A. R. Emerging concepts for the dynamical organization of resting-state activity in the brain. *Nature Reviews Neuroscience* **12**, 43–56 (2011).

[14] Murray, J. *Chapter 2. Mathematical biology II: spatial models and biomedical applications* (New York: Springer, 2003).

[15] Noble, W. S. How does multiple testing correction work? *Nature Biotechnology* **27**, 1135–1137 (2009).

[16] Lévy, B. Laplace-beltrami eigenfunctions towards an algorithm that" understands" geometry. In *Shape Modeling and Applications, 2006. SMI 2006. IEEE International Conference on*, 13–13 (IEEE, 2006).

[17] Fienup, J. R. Invariant error metrics for image reconstruction. *Applied optics* **36**, 8352–8357 (1997).

[18] Tononi, G. & Koch, C. The Neural Correlates of Consciousness: An Update. *Annals of the New York Academy of Sciences* **1124**, 239–261 (2008).

[19] Alkire, M. T., Hudetz, A. G. & Tononi, G. Consciousness and Anesthesia. *Science* 1–5 (2008).

[20] Horovitz, S. G. *et al.* Decoupling of the brain's default mode network during deep sleep. *PNAS* 1–13 (2009).

[21] Samann, P. G. *et al.* Development of the Brain's Default Mode Network from Wakefulness to Slow Wave Sleep. *Cerebral Cortex* **21**, 2082–2093 (2011).

[22] Lyapunov, A. M. The general problem of the stability of motion. *International Journal of Control* **55**, 531–534 (1992).
